# Supplementary material for: Study on deformation law of coal pore mechanism characteristics under peak cluster landform
Source: PLoS One. 2025 Aug 28;20(8):e0330388. doi: 10.1371/journal.pone.0330388 (PMC12393722; doi:10.1371/journal.pone.0330388)
Supplement: S1 File — (DOCX) [file pone.0330388.s001.docx]

**Fig 3 was taken by these text.**

| LF1 | | | | LF2 | | | |
| --- | --- | --- | --- | --- | --- | --- | --- |
| P | V | P | V | P | V | P | V |
| 0.01371 | 3.47E-31 | 413.75298 | 0.03825 | 0.01375 | 2.39E-31 | 413.78127 | 0.03983 |
| 0.0206 | 5.18418E-4 | 318.08862 | 0.03295 | 0.02062 | 0.0025 | 318.08586 | 0.03395 |
| 0.0275 | 8.21389E-4 | 244.96587 | 0.02836 | 0.02751 | 0.00372 | 244.96035 | 0.02926 |
| 0.03783 | 0.00113 | 188.38794 | 0.02468 | 0.03784 | 0.00472 | 188.43555 | 0.02541 |
| 0.04129 | 0.00121 | 144.99591 | 0.02154 | 0.04128 | 0.00497 | 144.99522 | 0.02243 |
| 0.05159 | 0.00145 | 110.52351 | 0.0189 | 0.05161 | 0.0055 | 110.50695 | 0.01989 |
| 0.05849 | 0.00156 | 85.698 | 0.01687 | 0.05847 | 0.00575 | 85.67109 | 0.01794 |
| 0.07223 | 0.00176 | 66.42513 | 0.01522 | 0.07224 | 0.00617 | 66.40463 | 0.01639 |
| 0.08942 | 0.00199 | 50.43686 | 0.01373 | 0.08938 | 0.0065 | 50.54022 | 0.01508 |
| 0.11026 | 0.00217 | 39.41149 | 0.01264 | 0.11031 | 0.00682 | 39.4444 | 0.01417 |
| 0.13779 | 0.00251 | 29.72872 | 0.01166 | 0.13779 | 0.00714 | 29.58092 | 0.01321 |
| 0.17222 | 0.00286 | 22.72253 | 0.01077 | 0.17224 | 0.00745 | 22.78753 | 0.01252 |
| 0.20682 | 0.00314 | 17.97899 | 0.01012 | 0.20685 | 0.00771 | 17.95435 | 0.01199 |
| 0.25035 | 0.00319 | 13.74715 | 0.00949 | 0.29812 | 0.00792 | 13.78296 | 0.01149 |
| 0.37572 | 0.00354 | 10.28411 | 0.00895 | 0.41366 | 0.00809 | 10.30881 | 0.01104 |
| 0.47558 | 0.00389 | 8.26696 | 0.00854 | 0.52226 | 0.00819 | 8.2722 | 0.01075 |
| 0.57941 | 0.00399 | 6.19928 | 0.00798 | 0.60285 | 0.00823 | 6.14732 | 0.01045 |
| 0.65373 | 0.00409 | 4.85706 | 0.00764 | 0.76176 | 0.00837 | 4.82003 | 0.01019 |
| 0.80066 | 0.00423 | 3.43998 | 0.00721 | 0.98057 | 0.00848 | 3.46559 | 0.00993 |
| 1.02315 | 0.00443 | 2.74731 | 0.00685 | 1.23493 | 0.00861 | 2.73484 | 0.0097 |
| 1.19639 | 0.00456 | 2.07164 | 0.0066 | 1.52381 | 0.00869 | 2.05358 | 0.00952 |
| 1.50548 | 0.00477 | 1.6351 | 0.0064 | 1.87739 | 0.00878 | 1.65358 | 0.0094 |
| 1.86628 | 0.00491 | 1.26824 | 0.00622 | 2.3096 | 0.00885 | 1.31809 | 0.00929 |
| 2.26833 | 0.00503 | 0.98464 | 0.00608 | 2.87856 | 0.00894 | 0.99052 | 0.00919 |
| 2.88359 | 0.00519 | 0.7705 | 0.00596 | 3.57955 | 0.00901 | 0.7382 | 0.0091 |
| 3.5993 | 0.00533 | 0.56633 | 0.0058 | 4.41545 | 0.00911 | 0.59744 | 0.00905 |
| 4.40581 | 0.00546 | 0.45155 | 0.00569 | 5.50601 | 0.00923 | 0.42546 | 0.00898 |
| 5.50404 | 0.00561 | 0.34459 | 0.00558 | 6.84214 | 0.00935 | 0.32471 | 0.00889 |
| 6.81395 | 0.00574 | 0.19707 | 0.00541 | 8.27814 | 0.00949 | 0.17863 | 0.00876 |
| 8.30056 | 0.00591 | 0.1117 | 0.00525 | 10.32261 | 0.00969 | 0.10445 | 0.00859 |
| 10.32764 | 0.00614 | -- | -- | 13.11103 | 0.00994 | -- | -- |
| 13.0813 | 0.00643 | -- | -- | 16.18347 | 0.01023 | -- | -- |
| 16.19672 | 0.00678 | -- | -- | 19.98689 | 0.01057 | -- | -- |
| 19.97557 | 0.00714 | -- | -- | 24.81516 | 0.01102 | -- | -- |
| 24.8202 | 0.00764 | -- | -- | 31.0026 | 0.01152 | -- | -- |
| 31.0233 | 0.0082 | -- | -- | 38.52505 | 0.01234 | -- | -- |
| 38.53919 | 0.00928 | -- | -- | 47.47117 | 0.01313 | -- | -- |
| 47.47083 | 0.01001 | -- | -- | 59.19924 | 0.01414 | -- | -- |
| 59.18654 | 0.01116 | -- | -- | 72.98199 | 0.0153 | -- | -- |
| 72.99579 | 0.01243 | -- | -- | 90.89784 | 0.01681 | -- | -- |
| 90.9144 | 0.01408 | -- | -- | 101.9406 | 0.01774 | -- | -- |
| 101.9475 | 0.01505 | -- | -- | 113.01786 | 0.01854 | -- | -- |
| 112.99164 | 0.01599 | -- | -- | 137.80611 | 0.02066 | -- | -- |
| 137.81715 | 0.0182 | -- | -- | 172.43652 | 0.0232 | -- | -- |
| 172.43514 | 0.021 | -- | -- | 206.96274 | 0.02579 | -- | -- |
| 206.97171 | 0.02399 | -- | -- | 241.47723 | 0.02837 | -- | -- |
| 241.45446 | 0.0265 | -- | -- | 275.95998 | 0.03084 | -- | -- |
| 275.96412 | 0.02909 | -- | -- | 310.45308 | 0.03315 | -- | -- |
| 310.44963 | 0.03145 | -- | -- | 344.94618 | 0.03537 | -- | -- |
| 344.89857 | 0.03354 | -- | -- | 379.34268 | 0.03762 | -- | -- |
| 379.30887 | 0.03578 | -- | -- | 413.78127 | 0.03983 | -- | -- |
| 413.75298 | 0.03825 | -- | -- |  |  |  |  |

| LF3 | | | | LF4 | | | |
| --- | --- | --- | --- | --- | --- | --- | --- |
| P | V | P | V | P | V | P | V |
| 0.01376 | 3.47E-31 | 413.76333 | 0.04252 | 0.01374 | 3.77E-31 | 413.7723 | 0.03682 |
| 0.0206 | 0.00102 | 318.08655 | 0.03619 | 0.0206 | 9.94427E-4 | 318.08517 | 0.03285 |
| 0.0275 | 0.00151 | 244.96725 | 0.03083 | 0.02749 | 0.00156 | 244.96242 | 0.02947 |
| 0.03783 | 0.00192 | 188.40864 | 0.02677 | 0.03783 | 0.00218 | 188.42037 | 0.02651 |
| 0.04128 | 0.00204 | 144.98625 | 0.02333 | 0.04127 | 0.00234 | 144.97452 | 0.02402 |
| 0.0516 | 0.0023 | 110.50764 | 0.02048 | 0.05158 | 0.00271 | 110.52696 | 0.02192 |
| 0.05849 | 0.00245 | 85.70352 | 0.0183 | 0.05847 | 0.00294 | 85.6911 | 0.02029 |
| 0.07225 | 0.00263 | 66.39056 | 0.01647 | 0.0722 | 0.00331 | 66.32384 | 0.01892 |
| 0.08939 | 0.00284 | 50.498 | 0.01495 | 0.08937 | 0.00367 | 50.48564 | 0.01771 |
| 0.11029 | 0.00301 | 39.43681 | 0.01385 | 0.11026 | 0.00407 | 39.39286 | 0.0168 |
| 0.13778 | 0.00322 | 29.56422 | 0.01266 | 0.13777 | 0.00445 | 29.75411 | 0.01594 |
| 0.17222 | 0.00342 | 22.86046 | 0.01192 | 0.17221 | 0.00485 | 22.7651 | 0.01507 |
| 0.20686 | 0.00363 | 17.92171 | 0.01131 | 0.2068 | 0.00519 | 17.9725 | 0.01428 |
| 0.27712 | 0.0037 | 13.78454 | 0.01059 | 0.32857 | 0.00537 | 13.83733 | 0.01359 |
| 0.3429 | 0.00374 | 10.3244 | 0.01007 | 0.45185 | 0.00552 | 10.33351 | 0.01303 |
| 0.41524 | 0.0038 | 8.28386 | 0.00973 | 0.49529 | 0.00552 | 8.2259 | 0.01249 |
| 0.52647 | 0.00391 | 6.23962 | 0.00935 | 0.61952 | 0.00567 | 6.20553 | 0.01208 |
| 0.63964 | 0.00398 | 4.8155 | 0.00902 | 0.84648 | 0.00588 | 4.84984 | 0.01168 |
| 0.81688 | 0.0041 | 3.48402 | 0.00862 | 0.94793 | 0.00594 | 3.46793 | 0.01139 |
| 0.97908 | 0.00443 | 2.74379 | 0.00833 | 1.2605 | 0.00619 | 2.75168 | 0.0111 |
| 1.18908 | 0.0047 | 2.058 | 0.00807 | 1.51805 | 0.00632 | 2.08339 | 0.0108 |
| 1.53101 | 0.0051 | 1.6635 | 0.00785 | 1.891 | 0.00651 | 1.63398 | 0.01053 |
| 1.88033 | 0.00538 | 1.3048 | 0.00766 | 2.31425 | 0.00665 | 1.2378 | 0.01035 |
| 2.29659 | 0.00561 | 0.99619 | 0.00749 | 2.88345 | 0.00681 | 0.95332 | 0.01014 |
| 2.88158 | 0.00579 | 0.77023 | 0.00729 | 3.57121 | 0.00697 | 0.70791 | 0.00992 |
| 3.58118 | 0.00597 | 0.60626 | 0.00724 | 4.40033 | 0.00713 | 0.57889 | 0.00982 |
| 4.3929 | 0.00618 | 0.44353 | 0.00714 | 5.52455 | 0.00732 | 0.44576 | 0.00969 |
| 5.50045 | 0.00634 | 0.34706 | 0.0071 | 6.81786 | 0.00753 | 0.33907 | 0.00958 |
| 6.81715 | 0.00654 | 0.18313 | 0.00694 | 8.25978 | 0.00774 | 0.18351 | 0.00937 |
| 8.25944 | 0.00673 | 0.11597 | 0.00682 | 10.32523 | 0.00802 | 0.07305 | 0.00918 |
| 10.33158 | 0.00699 | -- | -- | 13.08019 | 0.00835 | -- | -- |
| 13.0855 | 0.00734 | -- | -- | 16.17595 | 0.00875 | -- | -- |
| 16.18926 | 0.00766 | -- | -- | 19.97143 | 0.00915 | -- | -- |
| 19.97516 | 0.00809 | -- | -- | 24.81481 | 0.00968 | -- | -- |
| 24.81109 | 0.00857 | -- | -- | 31.01798 | 0.01033 | -- | -- |
| 31.01253 | 0.00917 | -- | -- | 38.52925 | 0.01144 | -- | -- |
| 38.53091 | 0.01011 | -- | -- | 47.47876 | 0.01239 | -- | -- |
| 47.49346 | 0.01109 | -- | -- | 59.17744 | 0.01359 | -- | -- |
| 59.17461 | 0.01216 | -- | -- | 72.95853 | 0.01489 | -- | -- |
| 72.93162 | 0.01347 | -- | -- | 90.90405 | 0.01625 | -- | -- |
| 90.89853 | 0.01527 | -- | -- | 101.94543 | 0.01703 | -- | -- |
| 101.93163 | 0.01648 | -- | -- | 112.99371 | 0.01795 | -- | -- |
| 112.99371 | 0.01753 | -- | -- | 137.81508 | 0.0198 | -- | -- |
| 137.79507 | 0.01987 | -- | -- | 172.45308 | 0.02222 | -- | -- |
| 172.4448 | 0.02284 | -- | -- | 206.95239 | 0.0247 | -- | -- |
| 206.9793 | 0.02588 | -- | -- | 241.47861 | 0.0272 | -- | -- |
| 241.46205 | 0.02872 | -- | -- | 275.96067 | 0.02951 | -- | -- |
| 275.97723 | 0.0317 | -- | -- | 310.45929 | 0.03128 | -- | -- |
| 310.46619 | 0.03453 | -- | -- | 344.8965 | 0.03309 | -- | -- |
| 344.95101 | 0.03703 | -- | -- | 379.28955 | 0.03496 | -- | -- |
| 379.33302 | 0.03957 | -- | -- | 413.7723 | 0.03682 | -- | -- |
| 413.76333 | 0.04252 | -- | -- |  |  |  |  |

| LF5 | | | | LF6 | | | |
| --- | --- | --- | --- | --- | --- | --- | --- |
| P | V | P | V | P | V | P | V |
| 0.01374 | 3.18E-31 | 413.80059 | 0.03629 | 0.01375 | 3.3E-31 | 413.76333 | 0.04413 |
| 0.02061 | 7.02144E-4 | 318.08241 | 0.03043 | 0.02061 | 0.00459 | 318.0762 | 0.03812 |
| 0.02749 | 8.68441E-4 | 244.9638 | 0.02561 | 0.02751 | 0.00619 | 244.98312 | 0.03319 |
| 0.03783 | 9.97783E-4 | 188.41278 | 0.02165 | 0.03785 | 0.00653 | 188.39553 | 0.02926 |
| 0.04128 | 0.00103 | 144.99867 | 0.01851 | 0.04133 | 0.00662 | 145.00074 | 0.02597 |
| 0.0516 | 0.00111 | 110.52075 | 0.01583 | 0.05159 | 0.00681 | 110.52558 | 0.02323 |
| 0.05849 | 0.00116 | 85.70904 | 0.01375 | 0.05849 | 0.0069 | 85.698 | 0.02114 |
| 0.07218 | 0.00124 | 66.35578 | 0.01215 | 0.07221 | 0.00706 | 66.3722 | 0.01941 |
| 0.08942 | 0.00132 | 50.4704 | 0.0107 | 0.0894 | 0.00721 | 50.50103 | 0.01792 |
| 0.11027 | 0.00144 | 39.43937 | 0.00963 | 0.1103 | 0.00738 | 39.34718 | 0.01688 |
| 0.13778 | 0.00161 | 29.74121 | 0.00868 | 0.1378 | 0.00758 | 29.57064 | 0.01567 |
| 0.17223 | 0.00178 | 22.84845 | 0.00782 | 0.17226 | 0.00776 | 22.83465 | 0.01489 |
| 0.20681 | 0.00193 | 17.93489 | 0.00728 | 0.20688 | 0.00795 | 17.8926 | 0.01428 |
| 0.30025 | 0.00196 | 13.6865 | 0.00668 | 0.25638 | 0.008 | 13.8247 | 0.01368 |
| 0.33424 | 0.00196 | 10.16356 | 0.00615 | 0.34022 | 0.00806 | 10.25499 | 0.01304 |
| 0.40141 | 0.00202 | 8.26158 | 0.00582 | 0.42057 | 0.00814 | 8.30939 | 0.01267 |
| 0.52311 | 0.00214 | 6.21782 | 0.00538 | 0.50158 | 0.00823 | 6.22896 | 0.01225 |
| 0.59354 | 0.00222 | 4.74293 | 0.005 | 0.66676 | 0.00839 | 4.76003 | 0.01186 |
| 0.78537 | 0.00232 | 3.40848 | 0.00468 | 0.77626 | 0.00847 | 3.43643 | 0.01151 |
| 0.96801 | 0.00244 | 2.77749 | 0.0044 | 0.95505 | 0.0086 | 2.77515 | 0.0112 |
| 1.20471 | 0.00251 | 2.08905 | 0.00422 | 1.19589 | 0.00876 | 2.08573 | 0.01096 |
| 1.51109 | 0.00262 | 1.66581 | 0.00402 | 1.5122 | 0.0089 | 1.66698 | 0.01074 |
| 1.86412 | 0.0027 | 1.33275 | 0.00378 | 1.87841 | 0.00902 | 1.33293 | 0.01057 |
| 2.29514 | 0.00278 | 0.96768 | 0.00361 | 2.26925 | 0.00912 | 0.98649 | 0.01042 |
| 2.90896 | 0.00287 | 0.75855 | 0.00353 | 2.88615 | 0.00925 | 0.65384 | 0.01019 |
| 3.60041 | 0.00296 | 0.58942 | 0.00342 | 3.56955 | 0.00936 | 0.51819 | 0.01019 |
| 4.41058 | 0.00306 | 0.45054 | 0.00336 | 4.40924 | 0.0095 | 0.45106 | 0.01016 |
| 5.5288 | 0.00319 | 0.33899 | 0.00331 | 5.51841 | 0.00965 | 0.34488 | 0.0101 |
| 6.8481 | 0.00332 | 0.16311 | 0.00315 | 6.81664 | 0.00982 | 0.19427 | 0.01001 |
| 8.25909 | 0.00348 | 0.08782 | 0.00308 | 8.26344 | 0.01 | 0.08349 | 0.00987 |
| 10.3193 | 0.00369 | -- | -- | 10.3678 | 0.01022 | -- | -- |
| 13.09192 | 0.00398 | -- | -- | 13.08488 | 0.01051 | -- | -- |
| 16.17429 | 0.00431 | -- | -- | 16.18167 | 0.01084 | -- | -- |
| 19.98406 | 0.00467 | -- | -- | 19.97895 | 0.01123 | -- | -- |
| 24.80757 | 0.00514 | -- | -- | 24.80164 | 0.01172 | -- | -- |
| 31.03309 | 0.0057 | -- | -- | 31.02005 | 0.0123 | -- | -- |
| 38.51421 | 0.00658 | -- | -- | 38.53484 | 0.0132 | -- | -- |
| 47.46496 | 0.0074 | -- | -- | 47.47034 | 0.01416 | -- | -- |
| 59.18592 | 0.00854 | -- | -- | 59.16446 | 0.01525 | -- | -- |
| 72.95508 | 0.00976 | -- | -- | 72.93369 | 0.01654 | -- | -- |
| 90.8868 | 0.0112 | -- | -- | 90.88197 | 0.01815 | -- | -- |
| 101.95164 | 0.01229 | -- | -- | 101.93715 | 0.01925 | -- | -- |
| 112.99302 | 0.01329 | -- | -- | 112.97577 | 0.02026 | -- | -- |
| 137.80266 | 0.01547 | -- | -- | 137.80749 | 0.02235 | -- | -- |
| 172.44963 | 0.0184 | -- | -- | 172.43376 | 0.02564 | -- | -- |
| 206.97723 | 0.02115 | -- | -- | 206.96274 | 0.02822 | -- | -- |
| 241.47654 | 0.02401 | -- | -- | 241.4862 | 0.03105 | -- | -- |
| 275.95584 | 0.02633 | -- | -- | 275.95929 | 0.03397 | -- | -- |
| 310.46412 | 0.02878 | -- | -- | 310.46343 | 0.03642 | -- | -- |
| 344.89512 | 0.03137 | -- | -- | 344.8896 | 0.03913 | -- | -- |
| 379.29783 | 0.03415 | -- | -- | 379.30197 | 0.04155 | -- | -- |
| 413.80059 | 0.03629 | -- | -- | 413.76333 | 0.04413 | -- | -- |

| LF7 | | | | LF8 | | | |
| --- | --- | --- | --- | --- | --- | --- | --- |
| P | V | P | V | P | V | P | V |
| 0.01374 | 2.71E-31 | 413.75919 | 0.03918 | 0.01375 | 2.9E-31 | 413.7861 | 0.0377 |
| 0.0206 | 0.00354 | 318.08655 | 0.03293 | 0.02061 | 6.74755E-4 | 318.08172 | 0.03139 |
| 0.02749 | 0.00374 | 244.96587 | 0.02788 | 0.02749 | 9.05296E-4 | 244.96449 | 0.02632 |
| 0.03782 | 0.00388 | 188.40657 | 0.02371 | 0.03783 | 0.00114 | 188.42106 | 0.0223 |
| 0.04128 | 0.00392 | 145.00557 | 0.02034 | 0.0413 | 0.00119 | 145.01799 | 0.019 |
| 0.0516 | 0.00401 | 110.5104 | 0.01762 | 0.0516 | 0.00131 | 110.50005 | 0.01622 |
| 0.05849 | 0.00405 | 85.68489 | 0.0155 | 0.05848 | 0.00138 | 85.67454 | 0.01415 |
| 0.0722 | 0.00414 | 66.38669 | 0.01378 | 0.0722 | 0.00151 | 66.42375 | 0.01244 |
| 0.08942 | 0.00422 | 50.50241 | 0.01228 | 0.08942 | 0.00164 | 50.50559 | 0.01098 |
| 0.11028 | 0.00433 | 39.44778 | 0.01122 | 0.11029 | 0.00177 | 39.40624 | 0.00999 |
| 0.13779 | 0.00444 | 29.73866 | 0.0104 | 0.13781 | 0.00191 | 29.73803 | 0.00906 |
| 0.17225 | 0.00456 | 22.70066 | 0.00958 | 0.17226 | 0.00207 | 22.85211 | 0.00824 |
| 0.20684 | 0.00469 | 17.83602 | 0.00906 | 0.20685 | 0.00224 | 17.91537 | 0.00766 |
| 0.30452 | 0.00469 | 13.76233 | 0.00845 | 0.31531 | 0.0023 | 13.71651 | 0.00714 |
| 0.41455 | 0.00469 | 10.09712 | 0.00795 | 0.35275 | 0.0023 | 10.09553 | 0.00661 |
| 0.55178 | 0.00469 | 8.17291 | 0.00764 | 0.41162 | 0.00232 | 8.24136 | 0.00627 |
| 0.58926 | 0.00469 | 6.14417 | 0.00733 | 0.51445 | 0.00237 | 6.23678 | 0.00571 |
| 0.81931 | 0.00472 | 4.84944 | 0.0071 | 0.64812 | 0.00244 | 4.82461 | 0.00536 |
| 0.94247 | 0.00474 | 3.45087 | 0.0068 | 0.76 | 0.0025 | 3.40318 | 0.00505 |
| 1.18852 | 0.00481 | 2.72912 | 0.00656 | 1.0007 | 0.00274 | 2.76394 | 0.00487 |
| 1.49474 | 0.0049 | 2.08863 | 0.00629 | 1.19994 | 0.00284 | 2.05011 | 0.00468 |
| 1.92478 | 0.00505 | 1.63035 | 0.0062 | 1.51136 | 0.003 | 1.63467 | 0.00453 |
| 2.27578 | 0.00506 | 1.32023 | 0.00608 | 1.86964 | 0.00315 | 1.288 | 0.00428 |
| 2.89183 | 0.00514 | 0.98008 | 0.00604 | 2.26747 | 0.00325 | 1.00404 | 0.00416 |
| 3.58223 | 0.00525 | 0.78231 | 0.00588 | 2.90024 | 0.00337 | 0.76633 | 0.0041 |
| 4.42667 | 0.00534 | 0.61788 | 0.00588 | 3.60028 | 0.00348 | 0.60683 | 0.00406 |
| 5.51787 | 0.00545 | 0.43835 | 0.00575 | 4.40598 | 0.00357 | 0.45274 | 0.00396 |
| 6.85555 | 0.0056 | 0.37831 | 0.0057 | 5.4978 | 0.0037 | 0.35549 | 0.00391 |
| 8.26655 | 0.00573 | 0.19722 | 0.00562 | 6.83866 | 0.00386 | 0.19294 | 0.00384 |
| 10.3304 | 0.00595 | 0.08661 | 0.00552 | 8.30732 | 0.00399 | 0.09025 | 0.00375 |
| 13.08061 | 0.00622 | -- | -- | 10.35007 | 0.00421 | -- | -- |
| 16.19444 | 0.00654 | -- | -- | 13.08737 | 0.00449 | -- | -- |
| 19.97315 | 0.00695 | -- | -- | 16.18119 | 0.0048 | -- | -- |
| 24.80543 | 0.00738 | -- | -- | 19.99344 | 0.00521 | -- | -- |
| 31.0055 | 0.00797 | -- | -- | 24.79846 | 0.00565 | -- | -- |
| 38.53167 | 0.00894 | -- | -- | 31.00901 | 0.00624 | -- | -- |
| 47.47186 | 0.00968 | -- | -- | 38.54402 | 0.00715 | -- | -- |
| 59.18585 | 0.01076 | -- | -- | 47.47366 | 0.00797 | -- | -- |
| 72.95439 | 0.01202 | -- | -- | 59.18813 | 0.0091 | -- | -- |
| 90.89991 | 0.01366 | -- | -- | 72.93024 | 0.01026 | -- | -- |
| 101.95509 | 0.0147 | -- | -- | 90.88473 | 0.0119 | -- | -- |
| 113.01027 | 0.01564 | -- | -- | 101.93301 | 0.01286 | -- | -- |
| 137.82474 | 0.01792 | -- | -- | 112.98681 | 0.01391 | -- | -- |
| 172.43928 | 0.02102 | -- | -- | 137.80128 | 0.0162 | -- | -- |
| 206.98482 | 0.02373 | -- | -- | 172.44411 | 0.01925 | -- | -- |
| 241.48206 | 0.02651 | -- | -- | 206.94756 | 0.02214 | -- | -- |
| 275.96205 | 0.02944 | -- | -- | 241.46343 | 0.0248 | -- | -- |
| 310.45239 | 0.03176 | -- | -- | 275.9724 | 0.02767 | -- | -- |
| 344.93859 | 0.03419 | -- | -- | 310.44963 | 0.03024 | -- | -- |
| 379.26609 | 0.03681 | -- | -- | 344.91306 | 0.0328 | -- | -- |
| 413.75919 | 0.03918 | -- | -- | 379.27368 | 0.03534 | -- | -- |
|  |  |  |  | 413.7861 | 0.0377 | -- | -- |

| LF9 | | | |
| --- | --- | --- | --- |
| P | V | P | V |
| 0.01373 | 2.73E-31 | 413.76747 | 0.03743 |
| 0.02061 | 9.15894E-4 | 318.09069 | 0.03181 |
| 0.02749 | 0.00139 | 244.97898 | 0.02678 |
| 0.03783 | 0.00182 | 188.43969 | 0.02281 |
| 0.04128 | 0.00192 | 144.99729 | 0.0196 |
| 0.05159 | 0.00215 | 110.51592 | 0.01695 |
| 0.05848 | 0.00227 | 85.70145 | 0.01492 |
| 0.07221 | 0.00245 | 66.33349 | 0.01323 |
| 0.0894 | 0.00265 | 50.50234 | 0.01174 |
| 0.11029 | 0.00283 | 39.37119 | 0.01074 |
| 0.13779 | 0.00304 | 29.71223 | 0.00982 |
| 0.17222 | 0.00326 | 22.87557 | 0.00911 |
| 0.20686 | 0.00347 | 18.00783 | 0.00855 |
| 0.34089 | 0.00361 | 13.86389 | 0.00817 |
| 0.46977 | 0.00369 | 10.20593 | 0.0076 |
| 0.5245 | 0.00369 | 8.29704 | 0.00728 |
| 0.63668 | 0.00374 | 6.15287 | 0.00685 |
| 0.80415 | 0.00382 | 4.83323 | 0.00651 |
| 0.96997 | 0.00387 | 3.41639 | 0.00613 |
| 1.20473 | 0.00395 | 2.74086 | 0.00596 |
| 1.51001 | 0.00405 | 2.03549 | 0.00579 |
| 1.89173 | 0.00413 | 1.63742 | 0.00568 |
| 2.25406 | 0.00421 | 1.3346 | 0.00553 |
| 2.88343 | 0.00432 | 0.98856 | 0.00537 |
| 3.5656 | 0.00442 | 0.76461 | 0.0053 |
| 4.41566 | 0.00454 | 0.58833 | 0.00524 |
| 5.5083 | 0.00466 | 0.47124 | 0.00518 |
| 6.84128 | 0.00481 | 0.29489 | 0.00509 |
| 8.27959 | 0.00497 | 0.18185 | 0.00503 |
| 10.34993 | 0.00518 | 0.08469 | 0.00495 |
| 13.11793 | 0.00546 | -- | -- |
| 16.18761 | 0.00575 | -- | -- |
| 19.98019 | 0.00612 | -- | -- |
| 24.81337 | 0.00654 | -- | -- |
| 31.01377 | 0.00714 | -- | -- |
| 38.54009 | 0.00801 | -- | -- |
| 47.47255 | 0.00883 | -- | -- |
| 59.17599 | 0.00995 | -- | -- |
| 72.97647 | 0.01116 | -- | -- |
| 90.89439 | 0.0127 | -- | -- |
| 101.95992 | 0.01364 | -- | -- |
| 113.00682 | 0.01469 | -- | -- |
| 137.82336 | 0.01686 | -- | -- |
| 172.45032 | 0.01996 | -- | -- |
| 206.98827 | 0.02251 | -- | -- |
| 241.47171 | 0.02534 | -- | -- |
| 275.97378 | 0.02803 | -- | -- |
| 310.46481 | 0.03075 | -- | -- |
| 344.93238 | 0.03294 | -- | -- |
| 379.32681 | 0.03557 | -- | -- |
| 413.76747 | 0.03743 | -- | -- |

**Fig 4 was taken by these text.**

| LF1 | | LF2 | | LF3 | |
| --- | --- | --- | --- | --- | --- |
| Pore Diameter | Incremental | Pore Diameter | Incremental | Pore Diameter | Incremental |
| 91015.7 | 3.47E-31 | 90769.4 | 2.39E-31 | 90714.6 | 3.47E-31 |
| 60579.4 | 5.18418E-4 | 60535.7 | 0.0025 | 60575.5 | 0.00102 |
| 45381.1 | 3.02971E-4 | 45363.5 | 0.00122 | 45384.7 | 4.8459E-4 |
| 32986.1 | 3.09701E-4 | 32979.4 | 9.9842E-4 | 32984.8 | 4.1728E-4 |
| 30226 | 8.08E-5 | 30234.9 | 2.496E-4 | 30228 | 1.1442E-4 |
| 24189.7 | 2.3564E-4 | 24181.5 | 5.3156E-4 | 24187.3 | 2.6248E-4 |
| 21338 | 1.0772E-4 | 21342.1 | 2.5423E-4 | 21335.8 | 1.4807E-4 |
| 17277.5 | 2.0872E-4 | 17275.8 | 4.1601E-4 | 17272.2 | 1.8172E-4 |
| 13956.3 | 2.2218E-4 | 13962.4 | 3.3281E-4 | 13961.5 | 2.0864E-4 |
| 11318.1 | 1.8178E-4 | 11313.4 | 3.1431E-4 | 11315 | 1.6826E-4 |
| 9057.28 | 3.4337E-4 | 9056.86 | 3.1894E-4 | 9057.31 | 2.1537E-4 |
| 7246.39 | 3.501E-4 | 7245.48 | 3.1432E-4 | 7246.23 | 1.9518E-4 |
| 6034.08 | 2.8277E-4 | 6033.11 | 2.6347E-4 | 6033 | 2.0864E-4 |
| 4984.84 | 5.074E-5 | 4186.14 | 2.0958E-4 | 4503.3 | 7.474E-5 |
| 3321.51 | 3.4786E-4 | 3016.85 | 1.6611E-4 | 3639.38 | 4.254E-5 |
| 2624.09 | 3.4694E-4 | 2389.55 | 9.975E-5 | 3005.39 | 5.943E-5 |
| 2153.83 | 1.0388E-4 | 2070.1 | 4.284E-5 | 2370.44 | 1.0621E-4 |
| 1908.99 | 9.501E-5 | 1638.25 | 1.394E-4 | 1951.04 | 7.056E-5 |
| 1558.67 | 1.4305E-4 | 1272.69 | 1.0692E-4 | 1527.72 | 1.203E-4 |
| 1219.73 | 2.016E-4 | 1010.55 | 1.3486E-4 | 1274.62 | 3.286E-4 |
| 1043.1 | 1.3025E-4 | 818.972 | 8.081E-5 | 1049.51 | 2.7218E-4 |
| 828.947 | 2.0487E-4 | 664.729 | 8.183E-5 | 815.118 | 3.939E-4 |
| 668.686 | 1.4543E-4 | 540.335 | 7.409E-5 | 663.69 | 2.8457E-4 |
| 550.167 | 1.1672E-4 | 433.537 | 8.663E-5 | 543.397 | 2.2654E-4 |
| 432.78 | 1.6036E-4 | 348.636 | 7.345E-5 | 433.081 | 1.8532E-4 |
| 346.723 | 1.3593E-4 | 282.634 | 1.0202E-4 | 348.477 | 1.7676E-4 |
| 283.253 | 1.2873E-4 | 226.654 | 1.1383E-4 | 284.085 | 2.0653E-4 |
| 226.735 | 1.5156E-4 | 182.393 | 1.2766E-4 | 226.883 | 1.6368E-4 |
| 183.148 | 1.3257E-4 | 150.754 | 1.3267E-4 | 183.062 | 2.0152E-4 |
| 150.347 | 1.6589E-4 | 120.896 | 1.9851E-4 | 151.095 | 1.8847E-4 |
| 120.837 | 2.3157E-4 | 95.1837 | 2.5114E-4 | 120.791 | 2.6384E-4 |
| 95.4004 | 2.9287E-4 | 77.113 | 2.8997E-4 | 95.3693 | 3.4214E-4 |
| 77.0502 | 3.4537E-4 | 62.4388 | 3.418E-4 | 77.0857 | 3.2913E-4 |
| 62.4742 | 3.6922E-4 | 50.2902 | 4.538E-4 | 62.4755 | 4.2392E-4 |
| 50.28 | 4.9812E-4 | 40.2533 | 4.982E-4 | 50.2984 | 4.8098E-4 |
| 40.2265 | 5.5645E-4 | 32.3934 | 8.17E-4 | 40.2405 | 6.0272E-4 |
| 32.3816 | 0.00108 | 26.2888 | 7.965E-4 | 32.3885 | 9.3926E-4 |
| 26.289 | 7.2517E-4 | 21.0807 | 0.001 | 26.2764 | 9.758E-4 |
| 21.0852 | 0.00115 | 17.0996 | 0.00116 | 21.0894 | 0.00107 |
| 17.0963 | 0.00127 | 13.7292 | 0.00151 | 17.1114 | 0.00132 |
| 13.7268 | 0.00166 | 12.2421 | 9.298E-4 | 13.7292 | 0.0018 |
| 12.2412 | 9.698E-4 | 11.0421 | 8.05E-4 | 12.2431 | 0.00121 |
| 11.0447 | 9.411E-4 | 9.05589 | 0.00211 | 11.0445 | 0.00105 |
| 9.0552 | 0.0022 | 7.23722 | 0.00255 | 9.05664 | 0.00233 |
| 7.23726 | 0.0028 | 6.02987 | 0.00258 | 7.23685 | 0.00298 |
| 6.02961 | 0.00299 | 5.16802 | 0.00258 | 6.02939 | 0.00304 |
| 5.16851 | 0.00251 | 4.52224 | 0.00248 | 5.16834 | 0.00284 |
| 4.52218 | 0.00258 | 4.0198 | 0.00231 | 4.52196 | 0.00298 |
| 4.01984 | 0.00236 | 3.61783 | 0.00221 | 4.01963 | 0.00283 |
| 3.61833 | 0.00209 | 3.28979 | 0.00226 | 3.61778 | 0.0025 |
| 3.29008 | 0.00224 | 3.01598 | 0.0022 | 3.28988 | 0.00254 |
| 3.01619 | 0.00247 |  |  | 3.01612 | 0.00295 |

| LF4 | | LF5 | | LF6 | |
| --- | --- | --- | --- | --- | --- |
| Pore Diameter | Incremental | Pore Diameter | Incremental | Pore Diameter | Incremental |
| 90829.8 | 3.77E-31 | 90794.4 | 3.18E-31 | 90752.6 | 3.3E-31 |
| 60590.2 | 9.94427E-4 | 60550.9 | 7.02144E-4 | 60544.2 | 0.00459 |
| 45389.3 | 5.70333E-4 | 45399 | 1.66297E-4 | 45361.3 | 0.00159 |
| 32985.9 | 6.1421E-4 | 32992.9 | 1.29342E-4 | 32975 | 3.451E-4 |
| 30236.5 | 1.6086E-4 | 30234.2 | 3.0797E-5 | 30197.9 | 8.947E-5 |
| 24194.6 | 3.7291E-4 | 24187 | 8.007E-5 | 24188.3 | 1.9172E-4 |
| 21342 | 2.2667E-4 | 21337.3 | 4.927E-5 | 21337.2 | 8.947E-5 |
| 17284 | 3.656E-4 | 17290.2 | 8.007E-5 | 17282.7 | 1.5977E-4 |
| 13964.3 | 3.656E-4 | 13956.8 | 8.623E-5 | 13958.8 | 1.4699E-4 |
| 11318.6 | 3.9484E-4 | 11317.4 | 1.1086E-4 | 11313.9 | 1.7255E-4 |
| 9058.15 | 3.8754E-4 | 9057.9 | 1.7246E-4 | 9056.35 | 1.9811E-4 |
| 7246.61 | 3.9484E-4 | 7245.98 | 1.7246E-4 | 7244.7 | 1.8533E-4 |
| 6034.49 | 3.4367E-4 | 6034.46 | 1.5398E-4 | 6032.25 | 1.8533E-4 |
| 3798.16 | 1.7535E-4 | 4156.45 | 2.967E-5 | 4867.52 | 4.517E-5 |
| 2761.91 | 1.4904E-4 | 3733.74 | 0 | 3668.1 | 6.813E-5 |
| 2519.66 | 0 | 3108.92 | 6.083E-5 | 2967.3 | 7.334E-5 |
| 2014.39 | 1.5065E-4 | 2385.67 | 1.1995E-4 | 2488.05 | 9.363E-5 |
| 1474.3 | 2.1324E-4 | 2102.58 | 7.651E-5 | 1871.68 | 1.62E-4 |
| 1316.51 | 6.422E-5 | 1589.01 | 1.0282E-4 | 1607.64 | 7.584E-5 |
| 990.051 | 2.4176E-4 | 1289.19 | 1.2026E-4 | 1306.7 | 1.312E-4 |
| 822.081 | 1.3694E-4 | 1035.9 | 6.701E-5 | 1043.54 | 1.6408E-4 |
| 659.945 | 1.8626E-4 | 825.863 | 1.0403E-4 | 825.258 | 1.3696E-4 |
| 539.248 | 1.3943E-4 | 669.464 | 8.22E-5 | 664.369 | 1.1723E-4 |
| 432.8 | 1.6323E-4 | 543.74 | 8.283E-5 | 549.943 | 9.741E-5 |
| 349.45 | 1.5735E-4 | 429.006 | 9.113E-5 | 432.397 | 1.3087E-4 |
| 283.606 | 1.5982E-4 | 346.616 | 9.299E-5 | 349.612 | 1.1606E-4 |
| 225.893 | 1.8925E-4 | 282.947 | 9.907E-5 | 283.033 | 1.3785E-4 |
| 183.043 | 2.1472E-4 | 225.72 | 1.234E-4 | 226.145 | 1.5022E-4 |
| 151.088 | 2.1114E-4 | 182.234 | 1.3826E-4 | 183.075 | 1.6661E-4 |
| 120.865 | 2.7197E-4 | 151.101 | 1.5487E-4 | 151.021 | 1.8373E-4 |
| 95.4084 | 3.3629E-4 | 120.934 | 2.1357E-4 | 120.369 | 2.215E-4 |
| 77.1491 | 3.9863E-4 | 95.3229 | 2.8889E-4 | 95.3742 | 2.916E-4 |
| 62.4871 | 3.9536E-4 | 77.1568 | 3.278E-4 | 77.1216 | 3.313E-4 |
| 50.2909 | 5.3627E-4 | 62.4476 | 3.6172E-4 | 62.4636 | 3.899E-4 |
| 40.2334 | 6.5158E-4 | 50.3056 | 4.6914E-4 | 50.3176 | 4.871E-4 |
| 32.3899 | 0.0011 | 40.2138 | 5.6317E-4 | 40.2307 | 5.789E-4 |
| 26.2845 | 9.584E-4 | 32.4025 | 8.7913E-4 | 32.3852 | 8.944E-4 |
| 21.0884 | 0.00119 | 26.2922 | 8.1969E-4 | 26.2892 | 9.601E-4 |
| 17.105 | 0.0013 | 21.0854 | 0.00113 | 21.093 | 0.0011 |
| 13.7283 | 0.00136 | 17.1059 | 0.00123 | 17.1109 | 0.00128 |
| 12.2415 | 7.788E-4 | 13.7309 | 0.00144 | 13.7316 | 0.00161 |
| 11.0445 | 9.185E-4 | 12.2407 | 0.00109 | 12.2424 | 0.0011 |
| 9.05532 | 0.00185 | 11.0446 | 0.001 | 11.0462 | 0.00101 |
| 7.2365 | 0.00242 | 9.05613 | 0.00219 | 9.05581 | 0.00209 |
| 6.03017 | 0.00248 | 7.23665 | 0.00292 | 7.23732 | 0.00329 |
| 5.16799 | 0.0025 | 6.02945 | 0.00275 | 6.02986 | 0.00258 |
| 4.52223 | 0.00232 | 5.16803 | 0.00286 | 5.16782 | 0.00283 |
| 4.01972 | 0.00176 | 4.52231 | 0.00232 | 4.52226 | 0.00293 |
| 3.61836 | 0.00181 | 4.01966 | 0.00245 | 4.01967 | 0.00245 |
| 3.29025 | 0.00187 | 3.61837 | 0.00259 | 3.61843 | 0.0027 |
| 3.01605 | 0.00186 | 3.29018 | 0.00278 | 3.29014 | 0.00243 |
|  |  | 3.01585 | 0.00214 | 3.01612 | 0.00258 |

| LF7 | | LF8 | | LF9 | |
| --- | --- | --- | --- | --- | --- |
| Pore Diameter | Incremental | Pore Diameter | Incremental | Pore Diameter | Incremental |
| 90799.2 | 2.71E-31 | 90740.7 | 2.9E-31 | 90868.4 | 2.73E-31 |
| 60568.3 | 0.00354 | 60543.3 | 6.74755E-4 | 60564.7 | 9.15894E-4 |
| 45389.9 | 1.9424E-4 | 45390.7 | 2.30541E-4 | 45395.2 | 4.71186E-4 |
| 32996.5 | 1.4174E-4 | 32986 | 2.30544E-4 | 32991.1 | 4.2882E-4 |
| 30228.3 | 3.675E-5 | 30214.7 | 5.623E-5 | 30234.8 | 1.0059E-4 |
| 24184 | 8.925E-5 | 24186 | 1.1808E-4 | 24191.3 | 2.3295E-4 |
| 21336.5 | 4.724E-5 | 21339.2 | 6.747E-5 | 21340.6 | 1.2176E-4 |
| 17283.8 | 8.925E-5 | 17283.7 | 1.2933E-4 | 17282.2 | 1.8001E-4 |
| 13956.6 | 7.874E-5 | 13956.6 | 1.2933E-4 | 13959.3 | 2.0118E-4 |
| 11316.8 | 1.05E-4 | 11314.8 | 1.2933E-4 | 11315.7 | 1.8E-4 |
| 9056.86 | 1.1024E-4 | 9055.97 | 1.462E-4 | 9056.98 | 2.1177E-4 |
| 7245.21 | 1.2075E-4 | 7244.65 | 1.6306E-4 | 7246.15 | 2.1176E-4 |
| 6033.5 | 1.3649E-4 | 6033.17 | 1.6307E-4 | 6033 | 2.1177E-4 |
| 4098.08 | 0 | 3957.84 | 5.734E-5 | 3660.84 | 1.3926E-4 |
| 3010.37 | 0 | 3537.85 | 0 | 2656.55 | 8.466E-5 |
| 2261.7 | 0 | 3031.8 | 2.551E-5 | 2379.32 | 0 |
| 2117.82 | 0 | 2425.83 | 4.515E-5 | 1960.11 | 4.802E-5 |
| 1523.19 | 2.212E-5 | 1925.52 | 7.42E-5 | 1551.9 | 7.741E-5 |
| 1324.13 | 2.658E-5 | 1642.04 | 6.415E-5 | 1286.6 | 5.64E-5 |
| 1050.01 | 6.459E-5 | 1247.08 | 2.3218E-4 | 1035.89 | 8.118E-5 |
| 834.899 | 9.14E-5 | 1040.02 | 1.081E-4 | 826.457 | 9.392E-5 |
| 648.366 | 1.5517E-4 | 825.721 | 1.5404E-4 | 659.692 | 8.027E-5 |
| 548.365 | 9.63E-6 | 667.488 | 1.493E-4 | 553.649 | 7.904E-5 |
| 431.546 | 7.864E-5 | 550.375 | 9.841E-5 | 432.802 | 1.1487E-4 |
| 348.375 | 1.1148E-4 | 430.295 | 1.1938E-4 | 350 | 9.895E-5 |
| 281.918 | 8.84E-5 | 346.628 | 1.1368E-4 | 282.621 | 1.2266E-4 |
| 226.167 | 1.079E-4 | 283.242 | 9.49E-5 | 226.56 | 1.1633E-4 |
| 182.036 | 1.4607E-4 | 226.993 | 1.25E-4 | 182.416 | 1.5088E-4 |
| 150.965 | 1.3589E-4 | 182.486 | 1.5927E-4 | 150.727 | 1.5552E-4 |
| 120.804 | 2.1661E-4 | 150.223 | 1.3547E-4 | 120.576 | 2.0845E-4 |
| 95.405 | 2.7583E-4 | 120.575 | 2.1988E-4 | 95.134 | 2.8716E-4 |
| 77.0609 | 3.2082E-4 | 95.3562 | 2.7389E-4 | 77.0934 | 2.8825E-4 |
| 62.4818 | 4.016E-4 | 77.1239 | 3.1378E-4 | 62.4598 | 3.6561E-4 |
| 50.3099 | 4.3839E-4 | 62.4185 | 4.0791E-4 | 50.2938 | 4.2374E-4 |
| 40.2496 | 5.8266E-4 | 50.324 | 4.3631E-4 | 40.2388 | 5.964E-4 |
| 32.3878 | 9.7499E-4 | 40.245 | 5.9822E-4 | 32.3808 | 8.7449E-4 |
| 26.2884 | 7.394E-4 | 32.3775 | 9.0118E-4 | 26.288 | 8.1871E-4 |
| 21.0854 | 0.00108 | 26.2874 | 8.2851E-4 | 21.0889 | 0.00112 |
| 17.106 | 0.00126 | 21.0846 | 0.00113 | 17.1009 | 0.00121 |
| 13.729 | 0.00164 | 17.1116 | 0.00116 | 13.7297 | 0.00155 |
| 12.2403 | 0.00104 | 13.7312 | 0.00163 | 12.2397 | 9.368E-4 |
| 11.0429 | 9.447E-4 | 12.2429 | 9.608E-4 | 11.0432 | 0.00105 |
| 9.05467 | 0.00228 | 11.0452 | 0.00105 | 9.05478 | 0.00216 |
| 7.2371 | 0.0031 | 9.05623 | 0.00228 | 7.23664 | 0.00311 |
| 6.02923 | 0.00271 | 7.23688 | 0.00305 | 6.02913 | 0.00254 |
| 5.16791 | 0.00278 | 6.03032 | 0.00289 | 5.16813 | 0.00283 |
| 4.52221 | 0.00293 | 5.16831 | 0.00266 | 4.52202 | 0.0027 |
| 4.0198 | 0.00232 | 4.52204 | 0.00287 | 4.01964 | 0.00272 |
| 3.61792 | 0.00244 | 4.01984 | 0.00257 | 3.61798 | 0.00219 |
| 3.29046 | 0.00261 | 3.61818 | 0.00257 | 3.28993 | 0.00264 |
| 3.01615 | 0.00237 | 3.29039 | 0.00253 | 3.01609 | 0.00186 |
|  |  | 3.01595 | 0.00236 |  |  |

**Fig 5 was taken by these text.**

| LF1 | | | | LF2 | | | |
| --- | --- | --- | --- | --- | --- | --- | --- |
| Pressure | Volume | Pressure | Volume | Pressure | Volume | Pressure | Volume |
| 0.00141 | 1.6951 | 0.9908 | 14.3552 | 0.00115 | 1.1676 | 0.99147 | 9.6427 |
| 0.00679 | 3.3688 | 0.93789 | 12.6286 | 0.00696 | 2.4082 | 0.94156 | 8.2511 |
| 0.01304 | 4.1239 | 0.89294 | 12.1131 | 0.02078 | 3.2665 | 0.89361 | 7.9029 |
| 0.02569 | 4.918 | 0.84404 | 11.7388 | 0.05551 | 3.9346 | 0.84563 | 7.7396 |
| 0.05023 | 5.7389 | 0.79563 | 11.464 | 0.08508 | 4.4546 | 0.79332 | 7.5971 |
| 0.08056 | 6.4735 | 0.74687 | 11.2335 | 0.1003 | 4.6489 | 0.73963 | 7.5029 |
| 0.0999 | 6.8375 | 0.69645 | 11.0279 | 0.12402 | 4.9002 | 0.69376 | 7.4082 |
| 0.12575 | 7.4168 | 0.64583 | 10.8411 | 0.14775 | 5.1561 | 0.64235 | 7.3276 |
| 0.15443 | 7.7292 | 0.59663 | 10.6687 | 0.17726 | 5.4146 | 0.59391 | 7.2503 |
| 0.17446 | 8.0834 | 0.54664 | 10.5046 | 0.19923 | 5.5299 | 0.54554 | 7.1689 |
| 0.20068 | 8.2673 | 0.49711 | 10.337 | 0.22515 | 5.647 | 0.4964 | 7.0854 |
| 0.22598 | 8.3882 | 0.45386 | 9.9037 | 0.25112 | 5.7316 | 0.44759 | 6.8911 |
| 0.24849 | 8.5412 | 0.39273 | 9.6255 | 0.2766 | 5.7964 | 0.39651 | 6.7697 |
| 0.27343 | 8.6803 | 0.34788 | 9.4725 | 0.30122 | 5.8831 | 0.34611 | 6.6724 |
| 0.29963 | 8.7721 | 0.29125 | 9.2989 | 0.35387 | 6.0144 | 0.29026 | 6.5624 |
| 0.35011 | 9.0003 | 0.24173 | 9.1292 | 0.40499 | 6.0958 | 0.24029 | 6.4429 |
| 0.40289 | 9.1308 | 0.19413 | 8.9421 | 0.45416 | 6.2038 | 0.19266 | 6.3124 |
| 0.45088 | 9.32 | 0.14329 | 8.723 | 0.49775 | 6.305 | 0.14398 | 6.1668 |
| 0.50181 | 9.4768 | 0.09281 | 8.4484 | 0.55371 | 6.4031 | 0.09599 | 5.9789 |
| 0.55192 | 9.6252 | -- | -- | 0.60423 | 6.4779 | -- | -- |
| 0.60183 | 9.7587 | -- | -- | 0.65375 | 6.5758 | -- | -- |
| 0.65041 | 9.9481 | -- | -- | 0.70328 | 6.675 | -- | -- |
| 0.70079 | 10.1093 | -- | -- | 0.75378 | 6.7842 | -- | -- |
| 0.74947 | 10.301 | -- | -- | 0.80128 | 6.9069 | -- | -- |
| 0.79894 | 10.5197 | -- | -- | 0.85001 | 7.0907 | -- | -- |
| 0.8471 | 10.8307 | -- | -- | 0.89999 | 7.3543 | -- | -- |
| 0.89569 | 11.2602 | -- | -- | 0.94672 | 7.7601 | -- | -- |
| 0.94235 | 11.9516 | -- | -- | 0.98414 | 8.885 | -- | -- |
| 0.97996 | 13.3212 | -- | -- | 0.99147 | 9.6427 | -- | -- |
| 0.9908 | 14.3552 | -- | -- |  |  |  |  |

| LF3 | | | | LF4 | | | |
| --- | --- | --- | --- | --- | --- | --- | --- |
| Pressure | Volume | Pressure | Volume | Pressure | Volume | Pressure | Volume |
| 0.00157 | 2.3868 | 0.99248 | 21.4324 | 0.00249 | 0.988 | 0.9916 | 10.8622 |
| 0.00606 | 4.851 | 0.93616 | 19.1285 | 0.01123 | 1.8927 | 0.93997 | 9.2037 |
| 0.01012 | 6.0627 | 0.89591 | 18.6354 | 0.03052 | 2.6377 | 0.89265 | 8.7542 |
| 0.03013 | 8.2255 | 0.8442 | 18.2627 | 0.05932 | 3.3423 | 0.84524 | 8.4465 |
| 0.05358 | 10.2595 | 0.79601 | 17.9973 | 0.07464 | 3.4773 | 0.79585 | 8.191 |
| 0.09009 | 11.3769 | 0.74519 | 17.7689 | 0.0965 | 3.896 | 0.74517 | 7.9619 |
| 0.12524 | 13.1502 | 0.69498 | 17.5671 | 0.12539 | 4.2731 | 0.69562 | 7.7518 |
| 0.15546 | 13.7546 | 0.64496 | 17.371 | 0.14663 | 4.8066 | 0.6453 | 7.5662 |
| 0.17888 | 14.1724 | 0.59405 | 17.1949 | 0.17619 | 4.9137 | 0.59629 | 7.3983 |
| 0.20234 | 14.3705 | 0.54518 | 17.0052 | 0.20476 | 5.1409 | 0.5466 | 7.2319 |
| 0.22391 | 14.5819 | 0.49585 | 16.8069 | 0.22923 | 5.2698 | 0.49705 | 7.0685 |
| 0.24807 | 14.7982 | 0.44827 | 16.5183 | 0.25581 | 5.3249 | 0.45131 | 6.6711 |
| 0.28057 | 14.9509 | 0.39775 | 16.2871 | 0.2725 | 5.4013 | 0.39865 | 6.4361 |
| 0.30113 | 15.0757 | 0.34061 | 16.0562 | 0.30402 | 5.5105 | 0.34759 | 6.2882 |
| 0.35063 | 15.3648 | 0.29015 | 15.8202 | 0.34875 | 5.6126 | 0.29097 | 6.1364 |
| 0.40086 | 15.626 | 0.24202 | 15.5659 | 0.40138 | 5.7681 | 0.24728 | 6.0047 |
| 0.44814 | 15.8458 | 0.19377 | 15.2834 | 0.44833 | 5.92 | 0.19398 | 5.8349 |
| 0.50062 | 16.0957 | 0.14449 | 14.9514 | 0.50293 | 6.0553 | 0.14487 | 5.6504 |
| 0.54754 | 16.2287 | 0.09383 | 14.5212 | 0.55342 | 6.1741 | 0.09552 | 5.4392 |
| 0.60146 | 16.424 | -- | -- | 0.60308 | 6.3006 | -- | -- |
| 0.65214 | 16.5755 | -- | -- | 0.65148 | 6.4895 | -- | -- |
| 0.70142 | 16.7785 | -- | -- | 0.70185 | 6.6446 | -- | -- |
| 0.75138 | 16.9593 | -- | -- | 0.75062 | 6.8476 | -- | -- |
| 0.79989 | 17.2023 | -- | -- | 0.80002 | 7.0859 | -- | -- |
| 0.84941 | 17.463 | -- | -- | 0.84849 | 7.3874 | -- | -- |
| 0.89671 | 17.8527 | -- | -- | 0.89667 | 7.8073 | -- | -- |
| 0.94206 | 18.5259 | -- | -- | 0.94348 | 8.4928 | -- | -- |
| 0.98132 | 20.0486 | -- | -- | 0.98175 | 9.8638 | -- | -- |
| 0.99248 | 21.4324 | -- | -- | 0.9916 | 10.8622 | -- | -- |

| LF5 | | | | LF6 | | | |
| --- | --- | --- | --- | --- | --- | --- | --- |
| Pressure | Volume | Pressure | Volume | Pressure | Volume | Pressure | Volume |
| 0.00303 | 3.6555 | 0.99107 | 23.5393 | 0.00536 | 6.1275 | 0.99119 | 25.2156 |
| 0.00627 | 5.4591 | 0.94421 | 20.7264 | 0.01034 | 7.6121 | 0.9411 | 22.262 |
| 0.01325 | 7.3091 | 0.89217 | 19.9769 | 0.03396 | 10.3277 | 0.89395 | 21.4539 |
| 0.02956 | 9.09 | 0.84263 | 19.5731 | 0.05446 | 11.6118 | 0.84346 | 20.9853 |
| 0.05819 | 10.8376 | 0.79426 | 19.2764 | 0.08059 | 12.863 | 0.79443 | 20.641 |
| 0.0785 | 11.774 | 0.74481 | 19.0236 | 0.10428 | 13.7927 | 0.74103 | 20.3664 |
| 0.09787 | 12.6244 | 0.69551 | 18.8011 | 0.12276 | 14.6483 | 0.69503 | 20.1308 |
| 0.1151 | 13.9576 | 0.64539 | 18.592 | 0.15148 | 15.6029 | 0.64422 | 19.8971 |
| 0.17565 | 14.8965 | 0.59505 | 18.3844 | 0.18 | 16.1318 | 0.59538 | 19.6787 |
| 0.20034 | 15.6716 | 0.54548 | 18.1789 | 0.2025 | 16.4791 | 0.54658 | 19.4564 |
| 0.22341 | 15.8115 | 0.49582 | 17.9729 | 0.22962 | 16.6339 | 0.49609 | 19.2267 |
| 0.25421 | 16.1344 | 0.44868 | 17.6733 | 0.25341 | 16.8461 | 0.44854 | 18.8578 |
| 0.27539 | 16.334 | 0.398 | 17.4202 | 0.27889 | 16.9948 | 0.39754 | 18.567 |
| 0.29986 | 16.5791 | 0.33974 | 17.1749 | 0.30376 | 17.1619 | 0.34037 | 18.2948 |
| 0.35216 | 16.892 | 0.29128 | 16.9255 | 0.34927 | 17.4352 | 0.29047 | 18.0089 |
| 0.39998 | 17.1165 | 0.2424 | 16.6525 | 0.39606 | 17.7988 | 0.24234 | 17.7018 |
| 0.44813 | 17.3402 | 0.19195 | 16.3456 | 0.44878 | 18.0823 | 0.19442 | 17.3667 |
| 0.49952 | 17.5444 | 0.14099 | 15.9769 | 0.50219 | 18.3148 | 0.14352 | 16.9708 |
| 0.5514 | 17.7169 | 0.09388 | 15.5345 | 0.55188 | 18.5614 | 0.09303 | 16.4499 |
| 0.59809 | 17.9438 | -- | -- | 0.60182 | 18.8142 | -- | -- |
| 0.64786 | 18.2271 | -- | -- | 0.65175 | 19.0553 | -- | -- |
| 0.70494 | 18.4162 | -- | -- | 0.7016 | 19.2804 | -- | -- |
| 0.74902 | 18.5838 | -- | -- | 0.75016 | 19.5829 | -- | -- |
| 0.79871 | 18.8035 | -- | -- | 0.79923 | 19.9042 | -- | -- |
| 0.84761 | 19.0633 | -- | -- | 0.84901 | 20.2368 | -- | -- |
| 0.89526 | 19.4531 | -- | -- | 0.89543 | 20.7915 | -- | -- |
| 0.94055 | 20.16 | -- | -- | 0.94414 | 21.6846 | -- | -- |
| 0.97785 | 21.7245 | -- | -- | 0.98108 | 23.6662 | -- | -- |
| 0.99107 | 23.5393 | -- | -- | 0.99119 | 25.2156 | -- | -- |

| LF7 | | | | LF8 | | | |
| --- | --- | --- | --- | --- | --- | --- | --- |
| Pressure | Volume | Pressure | Volume | Pressure | Volume | Pressure | Volume |
| 0.00304 | 3.797 | 0.99045 | 23.0492 | 0.00173 | 2.7867 | 0.99258 | 18.3888 |
| 0.00465 | 4.7452 | 0.94423 | 20.2749 | 0.00516 | 4.2686 | 0.93916 | 16.3888 |
| 0.01052 | 6.6511 | 0.89501 | 19.527 | 0.01075 | 5.6685 | 0.89447 | 15.9066 |
| 0.02589 | 8.4225 | 0.84429 | 19.1302 | 0.02403 | 6.9395 | 0.84629 | 15.6275 |
| 0.05107 | 10.1767 | 0.79491 | 18.8505 | 0.06154 | 9.0122 | 0.79409 | 15.3909 |
| 0.0894 | 11.4418 | 0.74569 | 18.6198 | 0.07709 | 9.5061 | 0.74441 | 15.1903 |
| 0.09977 | 12.8945 | 0.69553 | 18.4107 | 0.10705 | 9.9089 | 0.69379 | 15.025 |
| 0.1271 | 13.6377 | 0.64473 | 18.217 | 0.12255 | 10.787 | 0.64279 | 14.8731 |
| 0.15379 | 14.3572 | 0.59233 | 18.0389 | 0.15024 | 11.2127 | 0.59458 | 14.7226 |
| 0.17409 | 14.8197 | 0.54671 | 17.8584 | 0.17681 | 11.8297 | 0.54524 | 14.5588 |
| 0.20294 | 15.2943 | 0.49715 | 17.6742 | 0.19966 | 12.0911 | 0.49569 | 14.3955 |
| 0.22579 | 15.5106 | 0.449 | 17.3981 | 0.22273 | 12.2791 | 0.44723 | 14.1615 |
| 0.2519 | 15.7656 | 0.39097 | 17.1558 | 0.24909 | 12.4575 | 0.39672 | 13.9692 |
| 0.27814 | 15.9812 | 0.34786 | 16.9508 | 0.27405 | 12.6484 | 0.33959 | 13.7675 |
| 0.30348 | 16.095 | 0.29252 | 16.7086 | 0.30067 | 12.7523 | 0.28949 | 13.5608 |
| 0.35115 | 16.3161 | 0.24185 | 16.4516 | 0.35259 | 12.9854 | 0.24114 | 13.3388 |
| 0.3986 | 16.5681 | 0.19189 | 16.1684 | 0.39975 | 13.2157 | 0.19389 | 13.0917 |
| 0.44991 | 16.7664 | 0.14371 | 15.8337 | 0.45226 | 13.4438 | 0.14546 | 12.7925 |
| 0.49593 | 16.964 | 0.09751 | 15.4191 | 0.49877 | 13.5841 | 0.09494 | 12.4182 |
| 0.54974 | 17.1702 | -- | -- | 0.5521 | 13.7928 | -- | -- |
| 0.59522 | 17.3511 | -- | -- | 0.60325 | 13.9255 | -- | -- |
| 0.64784 | 17.5954 | -- | -- | 0.65298 | 14.0806 | -- | -- |
| 0.70054 | 17.7879 | -- | -- | 0.70224 | 14.2465 | -- | -- |
| 0.74888 | 18.0087 | -- | -- | 0.75203 | 14.3824 | -- | -- |
| 0.79624 | 18.2322 | -- | -- | 0.80031 | 14.599 | -- | -- |
| 0.84741 | 18.555 | -- | -- | 0.85008 | 14.8719 | -- | -- |
| 0.89615 | 18.9698 | -- | -- | 0.89902 | 15.1877 | -- | -- |
| 0.94213 | 19.6351 | -- | -- | 0.94429 | 15.7832 | -- | -- |
| 0.97742 | 21.2032 | -- | -- | 0.98303 | 17.2165 | -- | -- |
| 0.99045 | 23.0492 | -- | -- | 0.99258 | 18.3888 | -- | -- |

| LF9 | | | |
| --- | --- | --- | --- |
| Pressure | Volume | Pressure | Volume |
| 0.00367 | 4.3848 | 0.99061 | 24.8433 |
| 0.00818 | 6.6762 | 0.94454 | 22.7945 |
| 0.02378 | 8.8921 | 0.89234 | 22.2486 |
| 0.04961 | 10.9047 | 0.84522 | 22.0354 |
| 0.09118 | 12.4954 | 0.79415 | 21.9049 |
| 0.11605 | 14.9143 | 0.74497 | 21.8263 |
| 0.16903 | 15.9439 | 0.69422 | 21.7725 |
| 0.19835 | 17.3679 | 0.64517 | 21.7384 |
| 0.23622 | 17.6368 | 0.59511 | 21.7024 |
| 0.25235 | 17.7593 | 0.54519 | 21.673 |
| 0.27556 | 17.9855 | 0.49574 | 21.635 |
| 0.29857 | 18.2362 | 0.4469 | 21.4926 |
| 0.35382 | 18.5609 | 0.39771 | 21.417 |
| 0.39997 | 18.8271 | 0.34003 | 21.3398 |
| 0.45161 | 19.0428 | 0.29791 | 21.2758 |
| 0.50014 | 19.2967 | 0.24372 | 21.1458 |
| 0.55049 | 19.5074 | 0.19398 | 21.01 |
| 0.60137 | 19.6873 | 0.14283 | 20.7992 |
| 0.65035 | 19.9079 | 0.09222 | 20.4882 |
| 0.69996 | 20.1221 | -- | -- |
| 0.74898 | 20.3803 | -- | -- |
| 0.79774 | 20.6702 | -- | -- |
| 0.84742 | 21.0181 | -- | -- |
| 0.89674 | 21.4025 | -- | -- |
| 0.94222 | 22.0957 | -- | -- |
| 0.98031 | 23.6259 | -- | -- |
| 0.99061 | 24.8433 | -- | -- |

**Fig 6 was taken by these text.**

| LF1 | | | LF2 | | | LF3 | | |
| --- | --- | --- | --- | --- | --- | --- | --- | --- |
| Diameter | Volume | dV(d) | Diameter | Volume | dV(d) | Diameter | Volume | dV(d) |
| 147.1912 | 0.01818 | 0 | 159.954 | 0.01155 | 0 | 150.731 | 0.02599 | 0 |
| 67.0102 | 0.01662 | 3.532E-5 | 81.1745 | 0.01056 | 2.115E-5 | 70.4658 | 0.02423 | 3.503E-5 |
| 27.5616 | 0.01438 | 7.624E-5 | 29.3763 | 0.00873 | 3.835E-5 | 27.5724 | 0.02175 | 7.549E-5 |
| 16.7863 | 0.0132 | 1.241E-4 | 17.3285 | 0.00807 | 6.817E-5 | 16.9832 | 0.02062 | 1.107E-4 |
| 12.1059 | 0.01244 | 1.797E-4 | 12.2954 | 0.00761 | 9.99E-5 | 12.2324 | 0.01994 | 1.375E-4 |
| 9.4771 | 0.01186 | 1.992E-4 | 9.6109 | 0.00727 | 1.11E-4 | 9.5337 | 0.01947 | 2.314E-4 |
| 7.7673 | 0.01146 | 2.743E-4 | 7.8705 | 0.00705 | 1.418E-4 | 7.807 | 0.019 | 2.442E-4 |
| 6.5618 | 0.01108 | 3.089E-4 | 6.6216 | 0.00684 | 1.924E-4 | 6.5848 | 0.01866 | 4.268E-4 |
| 5.6609 | 0.01076 | 5.586E-4 | 5.7077 | 0.00664 | 2.651E-4 | 5.6738 | 0.01822 | 3.894E-4 |
| 4.9657 | 0.01033 | 4.426E-4 | 4.9927 | 0.00643 | 2.367E-4 | 4.9387 | 0.01791 | 6.817E-4 |
| 4.3965 | 0.01006 | 6.642E-4 | 4.3878 | 0.00628 | 3.98E-4 | 4.3665 | 0.01745 | 6.245E-4 |
| 3.9217 | 0.00971 | 8.747E-4 | 3.9159 | 0.00605 | 7.247E-4 | 3.9054 | 0.01716 | 0.00154 |
| 3.527 | 0.00933 | 0.00148 | 3.5472 | 0.00579 | 7.908E-4 | 3.5092 | 0.01646 | 0.00178 |
| 3.1807 | 0.00881 | 8.331E-4 | 3.1993 | 0.0055 | 5.628E-4 | 3.1753 | 0.01585 | 0.00238 |
| 2.8666 | 0.00853 | 0.00243 | 2.8823 | 0.00531 | 0.00129 | 2.8722 | 0.01508 | 0.00313 |
| 2.6526 | 0.00783 | 0.00176 | 2.6651 | 0.00492 | 0.00226 | 2.6753 | 0.01419 | 0.00356 |
| 2.5194 | 0.00758 | 0.00372 | 2.5342 | 0.00462 | 0.00143 | 2.537 | 0.0138 | 0.00242 |
| 2.4002 | 0.00711 | 0.00498 | 2.4047 | 0.00444 | 0.00216 | 2.3941 | 0.0134 | 0.00668 |
| 2.2838 | 0.00655 | 0.00287 | 2.2783 | 0.00416 | 0.00348 | 2.2827 | 0.0126 | 0.0076 |
| 2.161 | 0.0062 | 0.00529 | 2.1641 | 0.00373 | 0.00426 | 2.1753 | 0.01181 | 0.00598 |
| 2.0522 | 0.00555 | 0.01781 | 2.0432 | 0.00329 | 0.00819 | 2.065 | 0.01115 | 0.0168 |
| 1.9379 | 0.00388 | 0.00849 | 1.9181 | 0.00215 | 0.01016 | 1.9391 | 0.00931 | 0.01834 |
| 1.8083 | 0.00273 | 0.022 | 1.8049 | 0.00102 | 0.00888 | 1.8071 | 0.0067 | 0.05499 |

| LF4 | | | LF5 | | | LF6 | | |
| --- | --- | --- | --- | --- | --- | --- | --- | --- |
| Diameter | Volume | dV(d) | Diameter | Volume | dV(d) | Diameter | Volume | dV(d) |
| 151.9407 | 0.01584 | 0 | 142.5804 | 0.02946 | 0 | 150.0659 | 0.02958 | 0 |
| 72.101 | 0.01447 | 3.091E-5 | 61.8828 | 0.02674 | 4.621E-5 | 70.4332 | 0.02734 | 4.747E-5 |
| 27.9945 | 0.01224 | 7.32E-5 | 27.006 | 0.02418 | 8.198E-5 | 28.0866 | 0.02411 | 9.021E-5 |
| 16.9386 | 0.01107 | 1.201E-4 | 16.7693 | 0.023 | 1.108E-4 | 16.8467 | 0.02262 | 1.662E-4 |
| 12.1946 | 0.01033 | 1.701E-4 | 12.1222 | 0.02233 | 1.39E-4 | 12.1972 | 0.02163 | 1.739E-4 |
| 9.5242 | 0.00977 | 2.21E-4 | 9.4637 | 0.02188 | 1.984E-4 | 9.4961 | 0.02105 | 3.049E-4 |
| 7.7993 | 0.00931 | 2.92E-4 | 7.8099 | 0.02147 | 2.574E-4 | 7.7886 | 0.02042 | 4.518E-4 |
| 6.584 | 0.00891 | 2.908E-4 | 6.5893 | 0.02115 | 3.188E-4 | 6.5835 | 0.0198 | 4.385E-4 |
| 5.6793 | 0.0086 | 5.561E-4 | 5.6125 | 0.02077 | 8.95E-4 | 5.6728 | 0.01935 | 6.708E-4 |
| 4.983 | 0.00818 | 4.097E-4 | 4.9369 | 0.02008 | 9.57E-4 | 4.9654 | 0.01882 | 9.476E-4 |
| 4.4102 | 0.00792 | 4.746E-4 | 4.3829 | 0.01952 | 7.249E-4 | 4.398 | 0.01823 | 0.00118 |
| 3.9169 | 0.00768 | 6.536E-4 | 3.9003 | 0.01914 | 0.00117 | 3.9152 | 0.01763 | 0.00122 |
| 3.5118 | 0.00737 | 0.00117 | 3.5062 | 0.01863 | 0.00174 | 3.4954 | 0.01707 | 0.002 |
| 3.1714 | 0.00697 | 0.00119 | 3.1769 | 0.01802 | 0.00203 | 3.1549 | 0.01631 | 0.00378 |
| 2.8745 | 0.00656 | 8.608E-4 | 2.8734 | 0.01739 | 0.00321 | 2.8753 | 0.01518 | 0.00292 |
| 2.6621 | 0.00634 | 0.00192 | 2.6583 | 0.01643 | 0.00687 | 2.6781 | 0.01442 | 0.00373 |
| 2.5354 | 0.00602 | 0.00301 | 2.5388 | 0.01554 | 0.00652 | 2.546 | 0.01392 | 0.00305 |
| 2.4266 | 0.00576 | 6.242E-4 | 2.4083 | 0.01483 | 0.00764 | 2.4214 | 0.01352 | 0.00611 |
| 2.3014 | 0.00568 | 0.00394 | 2.2767 | 0.01366 | 0.003 | 2.297 | 0.0128 | 0.00278 |
| 2.1748 | 0.00522 | 0.00685 | 2.163 | 0.01333 | 0.03128 | 2.1783 | 0.01244 | 0.01356 |
| 2.038 | 0.00429 | 0.00147 | 1.9622 | 0.00968 | 0.01074 | 2.0583 | 0.01099 | 0.01671 |
| 1.9188 | 0.00409 | 0.02817 | 1.7829 | 0.00662 | 0.09019 | 1.9239 | 0.00876 | 0.03351 |
| 1.8075 | 0.00127 | 0.01035 |  |  |  | 1.8117 | 0.00423 | 0.0475 |

| LF7 | | | LF8 | | | LF9 | | |
| --- | --- | --- | --- | --- | --- | --- | --- | --- |
| Diameter | Volume | dV(d) | Diameter | Volume | dV(d) | Diameter | Volume | dV(d) |
| 141.7465 | 0.02609 | 0 | 155.9608 | 0.02292 | 0 | 148.0561 | 0.03468 | 0 |
| 61.5029 | 0.02318 | 4.865E-5 | 76.3773 | 0.02152 | 2.914E-5 | 67.8382 | 0.03281 | 3.826E-5 |
| 27.5419 | 0.02062 | 7.228E-5 | 28.4758 | 0.0192 | 6.25E-5 | 27.6231 | 0.03031 | 7.724E-5 |
| 16.8426 | 0.01952 | 1.153E-4 | 17.2353 | 0.0182 | 8.253E-5 | 16.8985 | 0.02914 | 1.048E-4 |
| 12.0512 | 0.01881 | 1.738E-4 | 12.2735 | 0.01766 | 1.478E-4 | 12.0894 | 0.02848 | 1.978E-4 |
| 9.3991 | 0.01822 | 2.166E-4 | 9.5555 | 0.01716 | 2.064E-4 | 9.4384 | 0.02783 | 2.861E-4 |
| 7.7544 | 0.0178 | 3.26E-4 | 7.8278 | 0.01674 | 1.732E-4 | 7.7492 | 0.02725 | 3.867E-4 |
| 6.5361 | 0.01736 | 3.611E-4 | 6.6021 | 0.01649 | 3.42E-4 | 6.5513 | 0.02672 | 4.398E-4 |
| 5.5928 | 0.01697 | 6.958E-4 | 5.6938 | 0.01614 | 4.3E-4 | 5.6571 | 0.02627 | 6.429E-4 |
| 4.9081 | 0.01641 | 7.495E-4 | 4.9766 | 0.0158 | 4.482E-4 | 4.9545 | 0.02578 | 6.17E-4 |
| 4.3571 | 0.01599 | 9.039E-4 | 4.3834 | 0.01551 | 9.967E-4 | 4.3807 | 0.02539 | 0.001 |
| 3.8907 | 0.0155 | 0.00131 | 3.9131 | 0.01497 | 8.67E-4 | 3.9168 | 0.02487 | 0.00165 |
| 3.5085 | 0.01499 | 0.00134 | 3.5217 | 0.01463 | 0.00169 | 3.5198 | 0.02419 | 0.00145 |
| 3.1692 | 0.01448 | 0.00244 | 3.1775 | 0.01398 | 0.00228 | 3.1819 | 0.02364 | 0.00264 |
| 2.8802 | 0.01374 | 0.00224 | 2.8769 | 0.01328 | 0.00227 | 2.8749 | 0.02285 | 0.00303 |
| 2.6754 | 0.01313 | 0.00229 | 2.657 | 0.01261 | 0.00196 | 2.6552 | 0.02189 | 0.00754 |
| 2.5402 | 0.01282 | 0.00573 | 2.5225 | 0.01233 | 0.00545 | 2.5346 | 0.02097 | 0.00669 |
| 2.4083 | 0.01205 | 0.00735 | 2.3938 | 0.01164 | 0.00467 | 2.435 | 0.02017 | 0.00471 |
| 2.2887 | 0.0111 | 0.00707 | 2.2734 | 0.01103 | 0.00614 | 2.3033 | 0.0198 | 0.00418 |
| 2.1655 | 0.01032 | 0.01469 | 2.1641 | 0.01035 | 0.00974 | 2.1427 | 0.01903 | 0.05056 |
| 2.0499 | 0.00832 | 0.0218 | 2.048 | 0.0093 | 0.02326 | 1.9489 | 0.01205 | 0.01304 |
| 1.9396 | 0.00625 | 0.02568 | 1.9205 | 0.00641 | 0.01208 | 1.7852 | 0.0088 | 0.1128 |
| 1.8115 | 0.00303 | 0.02316 | 1.818 | 0.00483 | 0.06479 |  |  |  |

The rest of the pictures can be drawn from the above data as well as from the data in the manuscript by calculating and deducing from the formulas.
