# Supplementary material for: Study on deformation law of coal pore mechanism characteristics under peak cluster landform
Source: PLoS One. 2025 Aug 28;20(8):e0330388. doi: 10.1371/journal.pone.0330388 (PMC12393722; doi:10.1371/journal.pone.0330388)
Supplement: Attachment — Submitted filename: Response to Reviewers.docx [file pone.0330388.s003.docx]

**Reviewer#1:**

1. In Fig 1(a), the meaning is unclear, please explain the specific meaning of the figure.

Response and revise: thank you very much for your valuable feedback. In Fig. 1(a), the left figure shows the surface contour map of the study area, while the right figure shows the plan view of the underground 120910 working face of the study area based on the surface contour, and the upper and lower two lines are the 120910 return lane and 120910 transportation lane, so the coal samples of LF1-LF9 were taken from the 120910 working face based on the different contour lines. Detailed information can be found in section 2.1 of the revised manuscript.

2. There are many inconsistencies in the row spacing of the tables in the text, such as tables 4 and 5.

Response and revise: thank you very much for your valuable feedback. Based on your guidance, we have set the full text tables to be uniformly spaced.

3. On pages 6, 8, and 14, the formula formats are inconsistent, please keep unified.

Response and revise: thank you very much for your valuable feedback. Based on your guidance, we have set all formulas to a uniform size. The specific revisions are listed below:

$P\text{=-}\frac{2\sigma\cos\theta}{r}$ （1）

（2）

（3）

（4）

（5）

$y=y_{0}+A/(w\sqrt{\frac{p_{i}}{4\ln2}})e^{\frac{-4\ln2(x-x_{c})^{2}}{w^{2}}}$ （6）

4. In lines 288 to 296, for the incomplete reference of the fractal theory method, appropriate references must be added to ensure academic rigor.

Response and revise: thank you very much for your valuable feedback. Based on your guidance, the manuscript has added relevant references such as:

[42] Zuo T, Li XL, Wang JG, Hu QW, Tao ZH, Hu T. Insights into natural tuff as a building material: Effects of natural joints on fracture fractal characteristics and energy evolution of rocks under impact load. Eng. Fail. Anal., 2024, 108584. https://doi.org/10.1016/j.engfailanal.2024.108584.

[44] Wang L, Wu SW, Han SR, Hu B, Wang Q, Zhang KZ, Song T. Fractal Analysis of Coal Pore Structure Based on Low-Pressure Gas Adsorption and Its Influence on Methane Adsorption Capacity: A Perspective from Micropore Filling Model. Energy Fuels 2024 38(5), 4031-4046. https://doi.org/10.1021/acs.energyfuels.3c04724.

[46] Zhang JS, Ni XM, Liu XL, Su E. Influences of Different Acid Solutions on Pore Structures and Fractal Features of Coal. Fractal Fract. 2024, 8(2). https://doi.org/10.3390/fractalfract8020082.

[49] Pan HJ, Shao YC, Liu ZZ, Zuo QL, Su JT, Bai JL, Miao HY, Guo YQ, Zhang JJ. A Physical Parameter Characterizing Heterogeneity of Pore and Fracture Structures in Coal Reservoirs. Processes 2024, 12(8), 1553. https://doi.org/10.3390/pr12081553.

[50] Zhu HR, Chen Y. Wang HC, Ma DM, Li GF, Ji CJ, Wang X, Shi JB. Differences in pore characteristics of different macroscopic coal rock components in low rank coal based on multi scale joint characterization. J. Xi’an Univ. Sci. Technol., 2025, 45(2), 404-418. https://doi.org/10.13800/j.cnki.xakjdxxb.2025.0218.

[51] Liu JJ, Zhang YL, Yang D, Gao ZY, Wang D. Joint characterization of middle and low rank coal pores and fractures based on pressed mercury-nitrogen adsorption-nuclear magnetic resonance method. J. Henan Polytech. Univ. Nat. Sci., 2025, 44(2), 19-31. https://doi.org/10.16186/j.cnki.1673-9787.2024010059.

5. In line 400, the author uses Gaussian function to analyze the relationship between vertical principal stress and pore structure and fractal dimension. Please explain why Gaussian function is used instead of other functions and explain its necessity.

Response and revise: thank you very much for your valuable feedback. We believe that the use of Gaussian function can be well matched with the statistical characteristics of the data, and the Gaussian function can transform the complex stress-pore structure relationship into interpretable parameters through a concise mathematical form, which provides a highly efficient tool for quantitatively revealing the control law of the principal stresses on the physical properties of coal reservoirs. Other functions, either due to lack of flexibility or vague physical meaning, are difficult to replace the analytical effect of Gaussian function under the same conditions. For example, although high-order polynomials can flexibly fit complex curves, they easily lead to overfitting phenomena and the physical meaning of the parameters is unclear, while the exponential function is only applicable to monotonically increasing or decreasing processes, and cannot describe the peak phenomena presented in the experimental results. Therefore, Gaussian function is used in this study to analyze the relationship between vertical principal stress and pore structure and fractal dimension.

Based on your guidance, we have added the description of choosing Gaussian function in section 4.1 of the revised manuscript. The specific descriptions are listed below:

According to the experimental results, the vertical principal stresses show a relatively symmetric peak phenomenon with respect to the pore structure and fractal dimension, which cannot be reflected by basic functions such as polynomial and exponential functions, for example, while the Gaussian function can naturally fit this statistical property, and its bell curve can effectively describe the concentration trend and the degree of discretization of the variables around the peak value.

**Reviewer #2:**

1. Page 4, what are the special conditions of the Peak Cluster landform? Please provide a detailed description in the text.

Response and revise: thank you very much for your valuable feedback.As a key component of the karst landform system, the peaked landforms are known for their outstanding uniqueness and structural complexity. The formation of these landforms is mainly attributed to the long-term influence of karst, and is characterized by a unique landscape consisting of isolated peaks and pillars with interspersed valleys and basins. In Guizhou, the vertical difference of the peaked landforms is especially significant, forming a typical steep topography, and the formation mechanism of this vertical height difference is closely related to the karst action and the regional geological structure. Under the influence of continuous water erosion, the difference in elevation between the peaks and gullies can be hundreds or even thousands of meters, resulting in high variability in the overlying rock stress.

Based on your guidance, we have added descriptions to the paper. Detailed information can be found in the introduction of the revised manuscript. The specific descriptions are listed below:

But the geological conditions of coal reservoirs, as an important factor restricting the development of coalbed methane, compared with other karst landforms, the peak cluster landforms between the peaks of the peak district is highly undulating., with a vertical height difference of hundreds of meters and steep slopes, resulting in high variability of the rock stresses overlying the coal reservoirs, which not only influences the complexity of the coal's pore structure, but also controls the gas transport channels of the coal reservoirs.

2. Page 6, formulas should be cited in the text and it should be ensured that the line height of formulas in the text remains consistent.

Response and revise: thank you very much for your valuable feedback. Based on your guidance, we have set all formulas to a uniform line height and added references to Eq. (1), (4), (5) and (6) in the text. The specific descriptions are listed below:

According to Eq. (1) Washburn's formula.

$P\text{=-}\frac{2\sigma\cos\theta}{r}$ （1）

（4）

Based on Eq. (4) the data related to ln(*V*/*V*_m_) and ln[ln(*P*_0_/*P*)] are fitted, as shown in Fig 9, the slope of the fitted line segment in the graph is the fractal dimension value.

Menger model for mesopore and macropore fractal dimension *D* is calculated as shown in Eq.(5).

（5）

$y=y_{0}+A/(w\sqrt{\frac{p_{i}}{4\ln2}})e^{\frac{-4\ln2(x-x_{c})^{2}}{w^{2}}}$ （6）

Based on Eq. (6), the vertical principal stress is fitted to the adsorption pore SSA and pore volume, and the fitting plot is shown in Fig 12.

3. On pages 20 and 21, Figure and Fig are not consistent in the text, and abbreviations and acronyms are not consistent.

Response and revise: thank you very much for your valuable feedback. Based on your guidance, we have harmonized the Figure with the Fig representation on pages 20 and 21.

4. On pages 303-448, change the canonical terms such as fractal dimension D to italics in the text as requested.

Response and revise: thank you very much for your valuable feedback. Based on your guidance, we have changed all relevant canonical terms covered in the text to italics.

5. On page 8, in the formula for calculating vertical principal stresses, is the value of stratum density taken into account in the field measurement data? It is recommended that the source of the density data be stated.

Response and revise: thank you very much for your valuable feedback. The stratum density of coal rock seams involved in this paper is taken based on the basic geological characteristics of the study area, so the stratum density value in the vertical principal stress calculation formula takes into account the geological borehole data and field measurement data of the study area. Based on your guidance, we have added relevant descriptions in the paper. Detailed information can be found in section 3.1 of the revised manuscript. The specific revisions are listed below:

Based on the geological borehole data and field measurements collected in the study area, the rock density for each layer of coal rock has been recorded, in which the average density of sandstone is 2500kg/m^3^, the average density of limestone is 2650kg/m^3^, the rock density of mudstone is 2300kg/m^3^, and the average density of coal is 1300kg/m^3^.

6. Page 14 and 15, pore systems can be described by a variety of fractal models. The authors have used the Frenkel-Halsey-Hill model and the Menger model to analyze pore structure complexity. Please explain the superiority of the two models in the description of pore structure complexity.

Response and revise: thank you very much for your valuable feedback. The Frenkel-Halsey-Hill model is based on the multilayer adsorption theory and quantifies the fractal characteristics of the pore surfaces through adsorption isotherm data.The FHH model calculates the fractal dimension (*D*) through the relationship between the relative pressure in the adsorption isotherm and the amount of adsorption. As *D* approaches 2, the surface is smooth; as *D* approaches 3, the pores exhibit a highly irregular layered structure, and this correlation provides a direct tool for quantifying the geometric complexity of the pore surface. In addition, the Frenkel-Halsey-Hill model not only reflects the surface fractal but also indirectly reveals the pore connectivity by analyzing the differences in the adsorption-desorption hysteresis loop. And the value of fractal dimension *D* obtained by the Menger model is directly related to the geometric complexity of pore network such as pore inhomogeneity, and can explain the reason for the in-and-out Hg hysteresis in the Hg compression curve. When *D* is close to 2, adsorption within the pore space is mainly dominated by open fractures; when *D* is close to 3, volume filling of isolated pores with poor connectivity within the pore space is a significant feature, and this correlation can intuitively explain the fractal behaviors of the different pressure segments in the piezomercury curves. Detailed information can be found in section 3.3 of the revised manuscript.

7. On page 18, this paper categorizes pores into adsorption and percolation pores, so please elaborate in the text on the reasons for analyzing only adsorption pores and bring in relevant literature to deepen the reader's understanding.

Response and revise: thank you very much for your valuable feedback. It is generally believed that pore size greater than 100nm is seepage pore and less than 100nm is adsorption pore. Coal gas adsorption is dominated by physical adsorption, which mainly occurs in micropore and transition pore with a pore diameter of less than 100nm, which have a very high specific surface area in the pores, accounting for more than 80% of the total specific surface area, and provide a large number of adsorption sites for gas molecules, which directly determines the amount of adsorption of coal gas. The mesopore and macropore with a pore size larger than 100 nm are mainly used as a channel for gas transportation, with a smaller specific surface area and a weaker contribution to the adsorption amount. In addition the gas in the seepage pores mostly exists in the free state rather than in the adsorbed state, so in the study of the effect of pore structure on gas adsorption, it is often considered only the adsorption pores, but not the seepage pores. Based on your guidance, we have added relevant descriptions in the paper. Detailed information can be found in section 4.1 of the revised manuscript. The specific revisions are listed below:

Based on previous studies, this paper defines pore size greater than 100nm as seepage pore and pore size less than 100nm as adsorption pore[52]. Coal gas adsorption is dominated by physical adsorption, which occurs on the coal surface, and gas molecules are more easily adsorbed on the pore surface when the pores have a high specific surface area. In addition, the gas mainly permeates in the form of laminar or turbulent flow in the macropore and mesopore , and exists in the form of capillary condensate or physical adsorption film on the pore wall in the micropore and transition pore, which indicates that the seepage pore mainly serves as a gas transport channel and the seepage pore has a small specific surface area, which makes a limited contribution to the amount of adsorption, while the adsorption pore has a very high specific surface area in the pore, accounting for more than 80% of the total specific surface area, which provides a large number of adsorption sites for the gas molecules provide a large number of adsorption sites, which directly determines the amount of coal gas adsorption[53]. Therefore, in order to study the differences in the pore structure of coal under the influence of vertical principal stress, this section mainly focuses on the variation rules of specific surface area and pore volume of adsorption pores with vertical principal stress.

[52] Li ZW, Lin BQ, Hao ZY, Gao YB. Characteristics ofpore size distribution of coal and its impacts on gas adsorption. J. China Univ. Min. Technol., 2013, 42(6): 1047-1053. https://doi.org/10.13247/j.cnki.jcumt.2013.06.025.

[53] Yang H, Bi WY, Zhang YG, Yu JK, Yan JW, Lei DJ, Ma ZN. Effect of tectonic coal structure on methane adsorption, J. Environ. Chem. Eng., 2021, 9(6), 106294. https://doi.org/10.1016/j.jece.2021.106294.

8.It is suggested that the author supplement the latest research literature on the theoretical methods of fractal dimension.

Response and revise: thank you very much for your valuable feedback. Based on your guidance, the manuscript has added relevant references such as:

[42] Zuo T, Li XL, Wang JG, Hu QW, Tao ZH, Hu T. Insights into natural tuff as a building material: Effects of natural joints on fracture fractal characteristics and energy evolution of rocks under impact load. Eng. Fail. Anal., 2024, 108584. https://doi.org/10.1016/j.engfailanal.2024.108584.

[44] Wang L, Wu SW, Han SR, Hu B, Wang Q, Zhang KZ, Song T. Fractal Analysis of Coal Pore Structure Based on Low-Pressure Gas Adsorption and Its Influence on Methane Adsorption Capacity: A Perspective from Micropore Filling Model. Energy Fuels 2024 38(5), 4031-4046. https://doi.org/10.1021/acs.energyfuels.3c04724.

[46] Zhang JS, Ni XM, Liu XL, Su E. Influences of Different Acid Solutions on Pore Structures and Fractal Features of Coal. Fractal Fract. 2024, 8(2). https://doi.org/10.3390/fractalfract8020082.

[49] Pan HJ, Shao YC, Liu ZZ, Zuo QL, Su JT, Bai JL, Miao HY, Guo YQ, Zhang JJ. A Physical Parameter Characterizing Heterogeneity of Pore and Fracture Structures in Coal Reservoirs. Processes 2024, 12(8), 1553. https://doi.org/10.3390/pr12081553.

[50] Zhu HR, Chen Y. Wang HC, Ma DM, Li GF, Ji CJ, Wang X, Shi JB. Differences in pore characteristics of different macroscopic coal rock components in low rank coal based on multi scale joint characterization. J. Xi’an Univ. Sci. Technol., 2025, 45(2), 404-418. https://doi.org/10.13800/j.cnki.xakjdxxb.2025.0218.

[51] Liu JJ, Zhang YL, Yang D, Gao ZY, Wang D. Joint characterization of middle and low rank coal pores and fractures based on pressed mercury-nitrogen adsorption-nuclear magnetic resonance method. J. Henan Polytech. Univ. Nat. Sci., 2025, 44(2), 19-31. https://doi.org/10.16186/j.cnki.1673-9787.2024010059.

**Reviewer #3:**

1. The article has limited discussion on the correlation between the peak cluster landform conditions and the vertical principal stress. It is recommended to strengthen the connection between the two at appropriate locations to enhance reader comprehension.

Response and revise: thank you very much for your valuable feedback. In response to your guidance, we have added a link between peak cluster landform and vertical principal stresses to the revised manuscript, details of which can be found in the Introduction section and Section 3.1 of the revised manuscript. The specific revisions are listed below:

The characteristics of the peak cluster landform result in significant vertical variability in the study area, subjecting the coal bodies to different pressures from the overlying rocks. Furthermore, the principal stress in the vertical direction, which represents the pressure acting along this axis of ground stress, primarily results from the gravitational force exerted by the rocks above. Therefore, in order to quantitatively obtain the different overlying rock pressures borne by the coal body, this paper analyzes the change rule of the pore structure of the coal body under the peak cluster landform by utilizing the stress calculated from the vertical principal stress formula.

2. In Figure 7, there appears to be a missing data point for the LF4 coal sample, which results in an incomplete adsorption-desorption curve. It is advisable to conduct a thorough review and make the necessary corrections to ensure the integrity of the data presented.

Response and revise: thank you very much for your valuable feedback. Based on your guidance, we've refined the image.

3. The theories and conclusions regarding the fractal method presented in this article are incomplete. To enhance the academic rigor of the paper, it is essential to provide appropriate references that support the claims made.

Response and revise: thank you very much for your valuable feedback. Based on your guidance, the manuscript has added relevant references such as:

[42] Zuo T, Li XL, Wang JG, Hu QW, Tao ZH, Hu T. Insights into natural tuff as a building material: Effects of natural joints on fracture fractal characteristics and energy evolution of rocks under impact load. Eng. Fail. Anal., 2024, 108584. https://doi.org/10.1016/j.engfailanal.2024.108584.

[44] Wang L, Wu SW, Han SR, Hu B, Wang Q, Zhang KZ, Song T. Fractal Analysis of Coal Pore Structure Based on Low-Pressure Gas Adsorption and Its Influence on Methane Adsorption Capacity: A Perspective from Micropore Filling Model. Energy Fuels 2024 38(5), 4031-4046. https://doi.org/10.1021/acs.energyfuels.3c04724.

[46] Zhang JS, Ni XM, Liu XL, Su E. Influences of Different Acid Solutions on Pore Structures and Fractal Features of Coal. Fractal Fract. 2024, 8(2). https://doi.org/10.3390/fractalfract8020082.

[49] Pan HJ, Shao YC, Liu ZZ, Zuo QL, Su JT, Bai JL, Miao HY, Guo YQ, Zhang JJ. A Physical Parameter Characterizing Heterogeneity of Pore and Fracture Structures in Coal Reservoirs. Processes 2024, 12(8), 1553. https://doi.org/10.3390/pr12081553.

[50] Zhu HR, Chen Y. Wang HC, Ma DM, Li GF, Ji CJ, Wang X, Shi JB. Differences in pore characteristics of different macroscopic coal rock components in low rank coal based on multi scale joint characterization. J. Xi’an Univ. Sci. Technol., 2025, 45(2), 404-418. https://doi.org/10.13800/j.cnki.xakjdxxb.2025.0218.

[51] Liu JJ, Zhang YL, Yang D, Gao ZY, Wang D. Joint characterization of middle and low rank coal pores and fractures based on pressed mercury-nitrogen adsorption-nuclear magnetic resonance method. J. Henan Polytech. Univ. Nat. Sci., 2025, 44(2), 19-31. https://doi.org/10.16186/j.cnki.1673-9787.2024010059.

4. In Section 4.1, the author categorizes pores into percolation pores and adsorption pores but lacks specific reasons for this distinction. It is suggested that the author add appropriate content and references to enhance reader comprehension.

Response and revise: thank you very much for your valuable feedback. Based on your guidance, we have added relevant descriptions in the paper. Detailed information can be found in section 4.1 of the revised manuscript. The specific revisions are listed below:

Based on previous studies, this paper defines pore size greater than 100nm as seepage pore and pore size less than 100nm as adsorption pore[52]. Coal gas adsorption is dominated by physical adsorption, which occurs on the coal surface, and gas molecules are more easily adsorbed on the pore surface when the pores have a high specific surface area. In addition, the gas mainly permeates in the form of laminar or turbulent flow in the macropore and mesopore , and exists in the form of capillary condensate or physical adsorption film on the pore wall in the micropore and transition pore, which indicates that the seepage pore mainly serves as a gas transport channel and the seepage pore has a small specific surface area, which makes a limited contribution to the amount of adsorption, while the adsorption pore has a very high specific surface area in the pore, accounting for more than 80% of the total specific surface area, which provides a large number of adsorption sites for the gas molecules provide a large number of adsorption sites, which directly determines the amount of coal gas adsorption[53]. Therefore, in order to study the differences in the pore structure of coal under the influence of vertical principal stress, this section mainly focuses on the variation rules of specific surface area and pore volume of adsorption pores with vertical principal stress.

[52] Li ZW, Lin BQ, Hao ZY, Gao YB. Characteristics ofpore size distribution of coal and its impacts on gas adsorption. J. China Univ. Min. Technol., 2013, 42(6): 1047-1053. https://doi.org/10.13247/j.cnki.jcumt.2013.06.025.

[53] Yang H, Bi WY, Zhang YG, Yu JK, Yan JW, Lei DJ, Ma ZN. Effect of tectonic coal structure on methane adsorption, J. Environ. Chem. Eng., 2021, 9(6), 106294. https://doi.org/10.1016/j.jece.2021.106294.

5. In Section 4.2, the meanings and interpretations of the fractal dimensions D1 and D2 need further description to enhance reader understanding and readability.

Response and revise: thank you very much for your valuable feedback. Based on your guidance, we have further described the meaning of the *D*_1_ and *D*_2_ values in Section 4.2. The specific revisions are listed below:

In order to further clarify the relationship between vertical principal stress and pore structure complexity, based on the previous content, the fractal dimension *D* is utilized to represent the pore structure complexity. The fractal dimension *D*_1_ is taken into account as it reflects the roughness of the pore surface; a higher *D*_1_ value suggests that the pore surface is rougher. Additionally, the fractal dimension *D*_2_, which represents the inhomogeneity of the pore structure, is also examined, with a greater *D*_2_ value indicating a more pronounced inhomogeneity in the pore structure.

6. The article employs a combination of mercury intrusion porosimetry and low-temperature nitrogen adsorption experiments. Please explain the advantages of combining these two methods for pore structure characterization and the necessity of establishing a critical pore diameter.

Response and revise: thank you very much for your valuable feedback. Firstly, from the principles of MIP and LPGA-N_2_, the MIP is based on the Washburn equation, which infiltrates mercury into pores by applying pressure, which is sensitive to mesopore, macropore and fissures, and is especially suitable for characterizing open-type connecting pores and seepage channels, but is limited by surface tension and contact angle of mercury, which leads to the extremely low efficiency of mercury intrusion into micropore and transition pore, while LPGA-N_2_ based on the BET model and BJH model, through the adsorption-desorption isotherm of nitrogen at low temperature, it can accurately analyze the micropore and transition pore, but the sensitivity of the characterization of mesopore and macropore is low. Therefore, the combination of MIP and LPGA-N_2_ realizes the pore space coverage at full scale and makes up for the pore detection blind spot of a single method.

Secondly, from the analysis of the results of MIP and LPGA-N_2_, MIP can analyze the complex structural features such as pore connectivity, tortuosity, and ‘ink-bottle pores’ through the Hg-in and Hg-out curves, which can reflect the pore geometrical morphology, and calculate the pore volume and the specific surface area of the mesopore and macropore based on Washburn's equation. The adsorption and desorption isotherm hysteresis loop can distinguish the pore shapes such as slit pores, cylindrical pores, ink bottle pores, etc., and can accurately calculate the pore volume and specific surface area of micropore and transition pore based on the BET model. Therefore, by integrating the pore volume and specific surface area data from the two methods, the differentiated contributions of different pore sizes to the storage capacity and permeability can be quantitatively analyzed. Finally, from the fractal characteristics obtained from the MIP and the LPGA-N_2_, the fractal dimensions of mesopore and macropore can be calculated from the MIP data based on the Menger model, while the fractal dimensions of micropore and transition pores can be calculated from the LPGA-N_2_ data based on the FHH model. Overall, the combination of MIP and LPGA-N_2_ can provide a more comprehensive and reliable data base for the characterization of the pore structure of coal through the synergistic analysis of morphology, specific surface area and fractal features. Detailed information can be found in sections 3.1, 3.2 and 3.3 of the revised manuscript.

In addition, the critical pore size was established to reduce the error of the experimental results. Because the MIP and LPGA-N_2_ will lead to part of the data overlap when analyzing the pore structure of the coal body, resulting in a large error in the experimental data. Therefore, the intersection point of the pore diameter versus pore volume curves determined by the two experiments was set as the critical pore diameter *r*_c_, so that the data repeated by the MIP and theLPGA-N_2_ were separated, and the LPGA-N_2_ data were used when the pore diameter *r* < *r*_c_, and the data from the MIP were used when *r* > *r*_c_, so that the pore structure of the coal could be analyzed in a comprehensive and reliable way. Detailed information can be found in section 3.2.3 of the revised manuscript.
