# Supplementary material for: Study on deformation law of coal pore mechanism characteristics under peak cluster landform
Source: PLoS One. 2025 Aug 28;20(8):e0330388. doi: 10.1371/journal.pone.0330388 (PMC12393722; doi:10.1371/journal.pone.0330388)
Supplement: Attachment — Submitted filename: Response_to_Reviewers_auresp_2.docx [file pone.0330388.s004.docx]

**Reviewer #4:**

This paper conducts pore characteristic analysis on 9 sample. A case about the variation in coal pore structure beneath peak cluster topography. The paper provides some information and data on the region, which can serve as a reference for readers or researchers. The authors have answered all the comments of three anonymous review experts. The biggest problem with this paper is that it did not provide any new insights, as there have been many studies on the pores and fractures of coal, and conventional methods were used in this paper. For data analysis, fractals were used. Suggest adding previous literature data in the sub parameter section for thorough discussion.

Response and revise: thank you very much for your valuable feedback. We fully understand and partially agree with your perspective. As you have pointed out, there is already a substantial body of literature in the field of research on coal pore and fracture structures, and this paper indeed employs analytical methods that are well-established and conventional in this field. However, we would like to emphasize the core objective and research context of this study. Our research is not aimed at proposing new theories or groundbreaking methods, but rather focuses on the specific geological context of peak cluster landform. We systematically characterize these landform using mature and reliable experimental methods such as high-pressure mercury intrusion and low-temperature nitrogen adsorption. Our primary contribution lies in providing detailed pore structure characteristics under the conditions of peak cluster landform, combined with fractal dimensions to gain a more comprehensive understanding. To more clearly highlight the value and positioning of this research, we have further elaborated on the uniqueness of the study area and the practical needs and application background of studying the pore structure in this region in the discussion section of the paper. The specific descriptions are listed below:

The geological conditions of coal reservoirs are an important factor in the development of CBM, and as a key component of the karst geomorphology system, the coal under this geomorphology has obvious vertical variability, which makes the pore structure of the coal more complex, and leads to the difficulty in clarifying the law of gas enrichment under this geomorphology. Therefore, the study of the multi-scale pore structure characteristics of coal and its complexity under the peak cluster landform can further provide reference for the study of the gas storage and enrichment law, which can not only deepen the gas storage law under special geological conditions, but also lay the foundation for the key technologies of resource development, disaster prevention and control in the similar topography, so as to guarantee the safety of mine mining.

According to reviewer 3, Please explain the advantages of combining these two methods for pore structure characterization and the necessity of establishing a critical pore diameter. This comment is very important, and it is recommended that the author further revise it.

Response and revise: thank you very much for your valuable feedback. It was previously mentioned in response to Reviewer 3's question that the mercury pressing experiment is based on the Washburn equation, which infiltrates mercury into pores by applying pressure, and it is sensitive to mesopors, macropore, and fissures, etc., and is especially suitable for characterizing open-type connected pores and seepage channels, but it is limited by the surface tension and the contact angle of mercury, which leads to the extremely low efficiency of mercury intrusion into micro- and small pores; whereas, the low-temperature nitrogen adsorption experiments are based on the BET model and the BJH model, through the adsorption-desorption isotherm of nitrogen at low temperature, it can accurately analyze the micro- and small pores, but the sensitivity of characterization of medium- and large-sized pores is low. Therefore, the combination of Hg-pressing experiments and low-temperature nitrogen adsorption realizes the full-scale coverage of pores and makes up for the pore size detection blind spot of a single method. The establishment of critical pore size is to reduce the error of the experimental results, because the pore structure of coal body analyzed by mercury pressure experiment and low temperature nitrogen gas adsorption experiment will lead to part of the data overlap, resulting in a large error in the experimental data, so the determination of the critical pore size can guarantee the accuracy of the analysis of the pore structure of the combination of the mercury pressure experiment and the low-temperature nitrogen gas adsorption experiment.

Based on your guidance, we have added descriptions to the paper. Detailed information can be found in section 3.2.3 of the revised manuscript. The specific descriptions are listed below:

Since the mercury pressure experiment is based on the pressure of mercury liquid to measure the pore size, at the beginning of the experiment, the mercury liquid was first pressed into the cracks of the coal samples, and then gradually pressed into the pores with the increase of pressure. However, as the pressure increases, some pores cannot resist the pressure of mercury liquid to form a large number of fissures, resulting in experimental deviation, and limited by the surface tension and contact angle of mercury, resulting in extremely low efficiency of mercury intrusion into micro and small pores; low-temperature liquid nitrogen adsorption is based on the cohesion of liquid nitrogen to fill the pores to detect the pore size, and is able to accurately analyze the micro and small pores, but has low sensitivity to the characterization of the medium and large pores, mainly because when measuring large pore sizes, the liquid nitrogen cannot coalesce.

The main reason is that when measuring the large pore size, the liquid nitrogen can not be coalesced with the increasing pressure, which makes the large pore size data of the measured coal samples have a large deviation. Therefore, characterizing the pore structure of coal by combining the two experiments not only realizes the full-scale coverage of pores and makes up for the blind spot of pore diameter detection of a single method, but also provides a more comprehensive and reliable data basis for the characterization of the pore structure of coal through the synergistic analysis of the morphology, specific surface area, and fractal features obtained from the two experiments. In addition, because the pore structure of coal can be partially overlapped when analyzing the pore structure of mercury pressure and low-temperature nitrogen adsorption experiments, resulting in a large error in the experimental data, based on the different sensitivities of the results of the two experiments for the characterization of multi-scale pores, a critical pore diameter was established as a pore diameter indicator to distinguish between the results of mercury pressure experiments and low-temperature nitrogen adsorption experiments, and the critical pore diameter was determined as the intersection point rc of the change curves of the measured pore diameters and the pore volumes of the two experiments.

The critical pore size was determined mainly as the intersection of two experimentally determined pore size versus pore volume curves *r*_c_. If the diameter of the pore is less than *r*_c_, data from the adsorption experiment with liquid nitrogen at low temperatures is utilized. Conversely, when the diameter of the pore is larger than *r*_c_, the data from the mercury intrusion experiment is utilized. Since some coal samples exhibit multiple intersections of the curves derived from both experiments, the last intersection point is selected as the critical pore diameter *r*_c_ for that particular coal sample. The determination of the critical pore size guarantees the accuracy of the pore structure analysis in the combination of mercury pressure experiments and low-temperature nitrogen adsorption experiments.

**Reviewer #5:**

After the previous round of revisions, the quality of the manuscript has been significantly improved. I believe that the manuscript has basically met the requirements for publication. But there are still a few issues that need further modification. The following comments and suggestions should guide the authors to revise the paper.

1. It is suggested that the authors further supplement the application significance and value of the research after the discussion section. This will be of great reference significance for researchers and engineers in related fields.

Response and revise: thank you very much for your valuable feedback. Based on your guidance, we have added descriptions to the paper. Detailed information can be found in the discussion of the revision. The specific descriptions are listed below:

The geological conditions of coal reservoirs are an important factor in the development of CBM, and as a key component of the karst geomorphology system, the coal under this geomorphology has obvious vertical variability, which makes the pore structure of the coal more complex, and leads to the difficulty in clarifying the law of gas enrichment under this geomorphology. Therefore, the study of the multi-scale pore structure characteristics of coal and its complexity under the peak cluster landform can further provide reference for the study of the gas storage and enrichment law, which can not only deepen the gas storage law under special geological conditions, but also lay the foundation for the key technologies of resource development, disaster prevention and control in the similar topography, so as to guarantee the safety of mine mining.

2.I noticed that some previous research literature related to this study was not cited in the article, such as previous research cases on coal pore structure and fractal characteristics. It is suggested that the authors further supplement the relevant literature.

Response and revise: thank you very much for your valuable feedback. Based on your guidance, the manuscript has added relevant references such as:

[30] Zhao DF, Zhang JM, Guan X, Liu DD, Wang QX, Jiao WW, Zhou XQ, Li YJ, Wang G, Guo YH. Comparing the Pore Networks of Coal, Shale, and Tight Sandstone Reservoirs of Shanxi Formation, Qinshui Basin: Inspirations for Multi-Superimposed Gas Systems in Coal-Bearing Strata. Appl. Sci. 2023; 13(7):4414. https://doi.org/10.3390/app13074414.

[44] Zhao DF, Guo YH, Wang G, Guan X, Zhou XQ, Liu J. Fractal Analysis and Classification of Pore Structures of High-Rank Coal in Qinshui Basin, China[J]. Energies. 2022; 15(18):6766. https://doi.org/10.3390/en15186766.

[58] Zhao DF, Guo YH, Wang G, Mao XX. Characterizing nanoscale pores and its structure in coal: Experimental investigation. Energ Explor Exploit, 2019, 37(4):1-28. https://doi.org/10.1177/0144598719831397.

3.The value and significance of new insights and findings in the manuscript are not highlighted in the abstract and conclusion.

Response and revise: thank you very much for your valuable feedback. Based on your guidance, we have added descriptions to the paper. Detailed information can be found in the Abstract and Conclusion of the revision. The specific descriptions are listed below:

Abstract:

In order to reveal the change rule of coal pore structure under the peak cluster landform, coal samples were taken from nine different mountain heights based on the vertical variability of the landform, and the pore structure of the coal samples was tested using a combination of high-pressure mercuric pressure method and low-temperature nitrogen adsorption experiments. The results show that compared with the traditional coal reservoir, the pore structure of coal under the peaked bush landscape, such as pore content, specific surface area and pore volume, changes with the change of vertical principal stress in a multi-peak state. The variations in the maximum and minimum values of vertical principal stress at each peak level are 1.04, 1.04, and 1.05 times, respectively. In terms of the adsorption pore volume, the differences between the maximum and minimum values are 2.30, 1.60, and 1.53 times, respectively. Notably, the degree of change between the peaks decreases as the peaks progress. Furthermore, with the increase in vertical principal stress, the degree of change in the specific surface area and pore volume of the corresponding adsorption pore between peaks also diminishes. It shows that the role of peak cluster landform conditions on coal pore structure is significant, and the extent of the role decreases with the increase of vertical principal stresses. Additionally, the vertical principal stress predominantly influences the fractal dimension *D*_1_, which is represented as pore surface roughness. The capacity of coal samples for gas adsorption is mainly influenced by the roughness of the pore surfaces and the volume of the adsorption pores. In summary, the degree of microcrack formation in the pores of coal samples is influenced to some extent by the vertical elevation difference characteristics of the peak cluster landform, which not only controls the characteristics of the pore structure, but also affects the gas adsorption capacity of the coal samples. These results highlight the influence of vertical principal stress on coal pore closure and structural changes under the peak cluster landform. The results of the study can provide a reference for further research on further gas storage and enrichment laws, and the mine can judge the risk of protrusion for the gas accumulation capacity of coal under the peaked cluster landform, so as to formulate effective gas prevention and control measures in advance.

Conclusion:

In this paper, the combination of high-pressure mercury pressure and low-temperature nitrogen adsorption testing method was used to quantitatively and comprehensively characterize the change rule of coal microporous structure under the influence of vertical height difference characteristics of the peak cluster landform, and based on the fractal theory, the fractal dimensions of the seepage pores and adsorption pores of the coal were respectively calculated, and then the change of the coal pore structure characteristics and its complexity under the peak cluster landform was investigated. The following conclusions were obtained:

(1) Influenced by the vertical elevation difference characteristics of the peak cluster landform, the coal is subjected to a gradual increase in vertical principal stresses in the overlying rock strata as the elevation of the mountain increases. Unlike individual techniques, the combined use of mercury injection and low-temperature nitrogen adsorption experiments provides better accuracy and thoroughness in analyzing the multi-scale pore structure of coal. The pore content obtained from this combined approach shows clear multi-peak variations that correspond to fluctuations in vertical principal stress.

(3) Compared with conventional coal reservoirs, the significant vertical height difference of the peak cluster landform leads to an obvious gradient of vertical principal stress, which exacerbates the non-homogeneous differentiation of pore structure. The fractal dimensions of adsorption pores, *D*_1_ and *D*_2_, show multi-peak variations with vertical principal stress, and the pore surface roughness of adsorption pores of coal samples is controlled by the vertical principal stress gradient under the peak cluster landform to a certain extent. In summary, the degree of closure of the pores of the coal samples under the crested clump topography varies in complexity with the vertical principal stress, which not only affects the pore structure characteristics of the coal samples, but also influences the gas adsorption capacity of the coal samples.

**Reviewer #6:**

1. The article is comprehensive in experimental design, covering coal sample analysis under different geological conditions and stress states. However, in order to enhance the reliability of the experiment and the statistical significance of the data, it is recommended to further discuss the possible sources of error in the experiment and consider increasing the number of repeated experiments. In addition, a more detailed error analysis should be provided for the processing and analysis of the experimental data to ensure the robustness of the conclusions.

Response and revise: thank you very much for your valuable feedback.We fully agree with the reviewers and recognize the importance of a thorough assessment of potential sources of error to enhance the reliability of the study. In this paper coal samples were drilled, cut, polished and screened in strict accordance with international standards to ensure that the geometric dimensions were within the standard range. The high-pressure mercury-pressure and low-temperature nitrogen adsorption equipment used in the experiments were strictly calibrated periodically, and zero-point calibration and performance verification were performed before and after each experiment, and the experiments were carried out in strictly controlled temperature and humidity environments to minimize the influence of environmental fluctuations on the experimental results. Through the above strict equipment management, sample preparation, environmental control and operation specification, we believe that the systematic errors in this study have been minimized. In addition, the experimental data in this study were obtained with highly accurate experimental instruments, and the study mainly involves the study of coal pore structure characteristics under 9 groups of different mountain heights, and no relevant replicated experiments have been conducted, so the data of this experiment are unique and do not provide a more detailed analysis of the error, in addition to the lack of samples, we can not carry out further replicated experiments. Based on your comments, we will take this part into full consideration in our subsequent studies, thank you again for your valuable feedback.

2. The method proposed in the article combines high-pressure mercury injection and low-temperature nitrogen adsorption experiments, and combines fractal theory to perform multi-scale quantitative characterization of the pore structure of coal samples, which shows significant innovation. It is recommended to further clarify the advantages of this method over traditional single characterization techniques and discuss in depth its potential application in the study of pore structure of other porous media (such as rocks, soils, etc.).

Response and revise: thank you very much for your valuable feedback. Based on your guidance, we have added descriptions to the paper. Detailed information can be found in section 3.2.3 of the revised manuscript. The specific descriptions are listed below:

Since the mercury pressure experiment is based on the pressure of mercury liquid to measure the pore size, at the beginning of the experiment, the mercury liquid was first pressed into the cracks of the coal samples, and then gradually pressed into the pores with the increase of pressure. However, as the pressure increases, some pores cannot resist the pressure of mercury liquid to form a large number of fissures, resulting in experimental deviation, and limited by the surface tension and contact angle of mercury, resulting in extremely low efficiency of mercury intrusion into micro and small pores; low-temperature liquid nitrogen adsorption is based on the cohesion of liquid nitrogen to fill the pores to detect the pore size, and is able to accurately analyze the micro and small pores, but has low sensitivity to the characterization of the medium and large pores, mainly because when measuring large pore sizes, the liquid nitrogen cannot coalesce.

The main reason is that when measuring the large pore size, the liquid nitrogen can not be coalesced with the increasing pressure, which makes the large pore size data of the measured coal samples have a large deviation. Therefore, characterizing the pore structure of coal by combining the two experiments not only realizes the full-scale coverage of pores and makes up for the blind spot of pore diameter detection of a single method, but also provides a more comprehensive and reliable data basis for the characterization of the pore structure of coal through the synergistic analysis of the morphology, specific surface area, and fractal features obtained from the two experiments.

3. Although the article compares and analyzes the experimental results and numerical simulation results in detail, there are some inconsistent results. For example, the simulated permeability of some coal samples is higher than the experimental value. It is recommended to conduct a more in-depth analysis of these inconsistencies, explore possible sources of simulation errors (such as model simplification, parameter setting, etc.), and propose corresponding improvement measures to improve the accuracy of the simulation.

Response and revise: thank you very much for your valuable feedback. However, no relevant numerical simulation experiments are involved in this study, so the next modification is not possible.

4. The article simplifies the formation of contact bridges when using DEM to simulate the compaction process of coal samples, which may affect the accuracy of the simulation results. It is recommended to further explore the specific impact of this simplification on the complexity of pore structure and permeability simulation results, and consider using more sophisticated geometric modeling methods (such as considering the irregularity of particle shape, dynamic changes in contact force, etc.) to improve the authenticity and accuracy of the simulation.

Response and revise: thank you very much for your valuable feedback. However, DEM experiments and related permeability simulation experiments were not involved in this study, so the next modification could not be made.

5. The abstract of the article needs to be revised, and the importance of the article should be highlighted. There are insufficient references, so more references need to be supplemented. There are too few references, which need to be supplemented to 30. The background and mechanism are not introduced clearly.

Mechanical behavior and fracture mechanism of high-temperature granite cooled with liquid nitrogen for geothermal reservoir applications. Physics of Fluids 2025; 37 (2): 026616. https://doi.org/10.1063/5.0253668

Response and revise: thank you very much for your valuable feedback. Based on your guidance, we have added descriptions to the paper. Detailed information can be found in the Abstract and Conclusion of the revision. The specific descriptions are listed below:

In order to reveal the change rule of coal pore structure under the peak cluster landform, coal samples were taken from nine different mountain heights based on the vertical variability of the landform, and the pore structure of the coal samples was tested using a combination of high-pressure mercuric pressure method and low-temperature nitrogen adsorption experiments. The results show that compared with the traditional coal reservoir, the pore structure of coal under the peaked bush landscape, such as pore content, specific surface area and pore volume, changes with the change of vertical principal stress in a multi-peak state. The variations in the maximum and minimum values of vertical principal stress at each peak level are 1.04, 1.04, and 1.05 times, respectively. In terms of the adsorption pore volume, the differences between the maximum and minimum values are 2.30, 1.60, and 1.53 times, respectively. Notably, the degree of change between the peaks decreases as the peaks progress. Furthermore, with the increase in vertical principal stress, the degree of change in the specific surface area and pore volume of the corresponding adsorption pore between peaks also diminishes. It shows that the role of peak cluster landform conditions on coal pore structure is significant, and the extent of the role decreases with the increase of vertical principal stresses. Additionally, the vertical principal stress predominantly influences the fractal dimension *D*_1_, which is represented as pore surface roughness. The capacity of coal samples for gas adsorption is mainly influenced by the roughness of the pore surfaces and the volume of the adsorption pores. In summary, the degree of microcrack formation in the pores of coal samples is influenced to some extent by the vertical elevation difference characteristics of the peak cluster landform, which not only controls the characteristics of the pore structure, but also affects the gas adsorption capacity of the coal samples. These results highlight the influence of vertical principal stress on coal pore closure and structural changes under the peak cluster landform. The results of the study can provide a reference for further research on further gas storage and enrichment laws, and the mine can judge the risk of protrusion for the gas accumulation capacity of coal under the peaked cluster landform, so as to formulate effective gas prevention and control measures in advance.

Based on your guidance, the manuscript has added relevant references such as:

[11] Mechanical behavior and fracture mechanism of high-temperature granite cooled with liquid nitrogen for geothermal reservoir applications. Physics of Fluids 2025; 37 (2): 026616. https://doi.org/10.1063/5.0253668.

6. When comparing the experimental and simulation data, the article mainly shows the average value, but does not provide statistics such as standard deviation and confidence interval. In order to more comprehensively evaluate the discreteness and consistency of the data, it is recommended to supplement these statistics. In addition, for the systematic deviation that may occur in the simulation, a sensitivity analysis should be conducted to determine the source of the deviation (such as model parameters, boundary conditions, etc.), and an effective correction method should be proposed to improve the reliability and practicality of the simulation results.

Response and revise: thank you very much for your valuable feedback. Since the relevant simulation data were not involved in this study, the next modification could not be made.

**Reviewer #7:**

1. The annotations on some charts (as shown in Figures 3 and 4) are not clear enough, especially regarding the units of the coordinate axes and the explanations of the legends. It is suggested to optimize the chart design and enhance the clarity of the chart to ensure that readers can understand the data intuitively.

Response and revise: thank you very much for your valuable feedback. Based on your guidance, we've refined the image.

2. Only 9 coal samples were collected in the text, which may be insufficient to cover different geological conditions under peak cluster landforms. It is suggested that the basis for sample selection be elaborated in detail in the methods section.

Response and revise: thank you very much for your valuable feedback. As a key component of the karst geomorphic system, the condition of peak cluster landform is known for its outstanding uniqueness and structural complexity, which is characterized by the unique landscape composed of isolated peaks and pillars, and intermittent valleys and basins, resulting in the peak cluster landform, compared with the other topographic features, showing obvious vertical variability among the mountain bodies. In Guizhou, the vertical variability of peak cluster landform is particularly significant, forming a typical steep topographic features, so in this study, we mainly consider the vertical variability of peak cluster landform, which is reflected in the difference in height between the mountains. Based on your guidance, we have further described the basis for sample selection in Section 2.1. The specific modifications are as follows:

In this study, based on the characteristics of vertical variability of peak cluster landform, which is specifically manifested in the different heights between mountains, the main basis for sample selection was the height of the overlying rock layer of the coal samples, and combined with the mountain contour map of Longfeng Coal Mine and the upper and lower control maps of the mine, nine groups of coal samples under different mountain heights were selected, and the height of mountains between each group of samples ranged from 10-25 m. The coal samples were collected at the same time.

3. Some research work can be useful in the introduction. Study on the degradation mechanism of mechanical properties of red sandstone under static and dynamic loading after different high temperatures.

Response and revise: thank you very much for your valuable feedback. Based on your guidance, the manuscript has added relevant references such as:

[8] Lin HX, Liu WD, Zhang D, Chen B, Zhang XS . Study on the degradation mechanism of mechanical properties of red sandstone under static and dynamic loading after different high temperatures. Sci. Rep., 2025, 15, 11611. https://doi.org/10.1038/s41598-025-93969-4.

4. In the data analysis section, using the Gaussian function to fit the data is innovative, but it only presents the fitting results and coefficients, lacking a detailed discussion of the goodness of fit test results.

Based on your guidance, we have added descriptions to the paper. Detailed information can be found in the section 4.1 of the revision. The specific descriptions are listed below:

While the RMSE value of pore volume was 0.0027 cc/g, the RMSE value of specific surface area was 4.5985 m²/g, indicating that the average deviation of the model predicted values from the measured values were both less than 12% of the range of the measured values. In addition, the residual analysis plots for adsorption pore SSA and pore volume are shown in Figure 13. The residual analysis of pore volume showed that the residuals showed a random distribution with the variation of vertical principal stresses, with no obvious trend or regular pattern, indicating that the Gaussian function model adequately described the main variations in the data, and no obvious systematic bias was found. While the residual analysis plot of the pore specific surface area showed a rising trend during the growth of the vertical principal stress, indicating that the Gaussian model may not be able to fully capture the subtle features of the pore structure changes in this stress interval, which may be related to the special changes in the specific surface area structure of the coal samples at this stage. Nevertheless, the overall goodness-of-fit index indicates that the Gaussian model is still an effective tool to describe the overall trend of this study.
